# Pathways to antisocial behavior: a framework to improve diagnostics and tailor therapeutic interventions

**DOI:** 10.3389/fpsyg.2023.993090

**Published:** 2023-02-09

**Authors:** Brenda De Wit-De Visser, Madeleine Rijckmans, Jeroen K. Vermunt, Arno van Dam

**Affiliations:** ^1^GGZ WNB, Research and Innovation, Halsteren, Netherlands; ^2^Tilburg School of Social and Behavioral Sciences, Tranzo Scientific Center for Care and Welfare, Tilburg University, Tilburg, Netherlands; ^3^Fivoor, Fivoor Science and Treatment Innovation, Poortugaal, Netherlands; ^4^Clinical and Forensic Psychology, Department of Developmental Psychology, Tilburg School of Social and Behavioral Sciences, Tilburg University, Tilburg, Netherlands; ^5^Department of Methodology and Statistics, Tilburg School of Social and Behavioral Sciences, Tilburg University, Tilburg, Netherlands

**Keywords:** antisocial personality disorder, antisocial behavior, psychotherapy, pathways, treatment, diagnostics, trust, reciprocity

## Abstract

The Antisocial Personality Disorder (ASPD), and antisocial behavior (ASB) in general, is associated with significant impact on individuals themselves, their environment, and society. Although various interventions show promising results, no evidence-based treatments are available for individuals with ASPD. Therefore, making informed choices about which treatment can be applied to an individual patient is complicated. Furthermore, contradictory findings on therapy effectiveness and underlying factors of ASB, such as cognitive impairments and personality traits, fuel the debate whether the conceptualization of ASPD in the DSM-5 is accurate and whether this population can be seen as homogeneous. A conceptual framework, based on the reciprocal altruism theory, is presented in which we propose different pathways to ASB. These pathways suggest underlying dynamics of ASB and provide an explanation for previous contradictory research outcomes. This framework is intended to serve as a clinically relevant model that provides directions for improving diagnostics and matching treatments to underlying dynamics in the antisocial population.

## Introduction

The Antisocial Personality Disorder (ASPD) is defined in the Diagnostic and Statistical Manual of Mental Disorders, Fifth Edition (DSM-5; American Psychiatric Association, 2013) as a ‘pervasive pattern of disregard for and violation of the rights of others’ (American Psychiatric Association, 2013) and encompasses a wide range of maladaptive behavior and interpersonal deficits. ASPD has a severe impact on individuals themselves, their environment, and society. The behaviors associated with ASPD, such as aggressiveness and criminality, might cause physical and emotional damage to others (Leone et al., 2004; Turner et al., 2010) and result in significant costs due to the use of the criminal justice system and health care facilities (Foster and Jones, 2005; Kiehl and Buckholtz, 2010). The relatively high prevalence of 1–3% in the general population in Western countries (Volkert et al., 2018) contributes to a substantial need for psychological interventions to reduce the impact of this mental disorder. It is therefore surprising that unlike the increase in evidence-based interventions that have become available for other cluster b and c personality disorders in the last decade, no evidence-based treatments are available for ASPD (Gibbon et al., 2020). However, several interventions show promising results (Van den Bosch et al., 2018), for example Dialectical Behavior Therapy (Wetterborg et al., 2020), Mentalization Based Treatment (Bateman et al., 2016) and Schema Focused Therapy (Bernstein et al., 2021). Furthermore, patients with ASPD who receive cognitive behavioral therapy aimed at ASPD-characteristics appear to benefit from it. Researchers reported reduced levels of substance abuse and psychiatric complaints (Davidson et al., 2009; Thylstrup et al., 2017). However, it is not clear whether these different treatment approaches are applicable to all ASPD patients or whether they target a specific psychopathological characteristic within the antisocial spectrum. This makes it hard for clinicians to make an informed choice for one of these treatment methods.

ASPD can co-occur with several mental disorders, such as cluster B personality disorders, substance abuse and attention deficit hyperactivity disorder (Tuvblad et al., 2009; Van Dam and Rijckmans, 2020). Various other mental illnesses are closely related to ASPD, such as Intermitted Explosive Disorder (IED), Conduct Disorder (CD), and psychopathy (Van Dam and Rijckmans, 2020). ASB can also (temporally) emerge in other mental disorders (Van Dam and Rijckmans, 2020), where it manifests itself differently. For example, ASB can manifest as a result of disinhibition in ADHD and substance use disorders (Saylor and Amann, 2016; Kraanen, 2020; Krueger et al., 2021). However, ASB can also arise from antagonistic personality traits, like difficulties in empathy in paranoid- and narcissistic personality disorders (Krueger et al., 2021), and lead to more proactive ASB. This illustrates that ASPD includes only a part of the broad population known with antisocial behavioral problems. In line with contemporary developments toward transdiagnostic reasoning (Insel et al., 2010; Kotov et al., 2017), we consider ASB as a transdiagnostic construct. This prevents further conceptual confusion by avoiding usage of classifications in describing the antisocial population. Furthermore, it meets the heterogeneity of the antisocial population and makes it possible to investigate possible underlying factors. ASB can be described as behavior that harms others and encompasses behavior that violates the rights of others (Tuvblad and Beaver, 2013). ASB includes more than just aggressive behaviors. Burt and colleagues (Burt et al., 2012) found evidence that ASB can be conceptualized as a construct of physical aggression (e.g., getting into physical fights, threatening others, hitting others when provoked), social aggression (e.g., blaming others, trying to hurt others’ feelings, being rude toward others), and rule-breaking behavior (e.g., burglary, drug dealing, stealing, non-compliance with agreements). Burt and Donnellan (2009) describe physical aggression as physical acts that may hurt others, but also as verbal aggression. Therefore, we prefer to use the term ‘physical and verbal aggression’ in our paper. Another widely used conceptualization is the difference between reactive and proactive aggressive behavior (Poulin and Boivin, 2000; Raine et al., 2006). Reactive aggression can evolve as a reaction to threat or provocation, whereas proactive aggression is more instrumental and calculated behavior. In the current paper, we use the conceptualization of Burt and Donnellan (2009) (physical and verbal aggression, social aggression and rule-breaking behavior) as well as the distinction between reactive and proactive aggression to describe ASB. This means that physical and verbal aggression, social aggression, and rule-breaking behavior may be reactive or proactive. In this light, ASB can be seen as a broad, heterogeneous construct which includes both overt and covert aspects.

To guide the development of evidence-based treatments and alleviate the burden of ASB, several researchers tried to get a better understanding of underlying factors of the dysfunctional behavioral patterns in this population. Within the antisocial spectrum, deficiencies in cognitive and affective functioning have been recurrently found. However, research outcomes show contradictory findings. Some researchers mentioned deficits in recognition of basic emotions (Chapman et al., 2018), others in recognition of sad faces (Blair et al., 2001; Dolan and Fullam, 2006), fearful faces (Blair et al., 2001; Montagne et al., 2005; Marsh and Blair, 2008), and anger and disgust (Jones Bartoli et al., 2007). Also, mentalizing abilities (the ability to make inferences about mental states of oneself and others) (Bateman and Fonagy, 2012) seem to vary in the antisocial population. Both hypermentalizing (overattribution of mental states) and hypomentalizing (underattribution of mental states) are found by several researchers (Dolan and Fullam, 2004; Abate et al., 2017; Newbury-Helps et al., 2017; Abi-Habib et al., 2020). Contradictory results are also reported regarding specific personality traits in the antisocial population. Within the antisocial population some individuals report low impulsivity (Hicks et al., 2004; Gray et al., 2019), while others seem to be highly impulsive (Loney et al., 2003; Maneiro et al., 2017; Gray et al., 2019). These contradictory findings fuel the debate whether the conceptualization of ASPD in the DSM-5 is accurate and comprehensive, and whether this population can be seen as homogeneous (Poythress et al., 2010; Mokros et al., 2015; Brazil et al., 2018).

In literature, the current classification of personality disorders in the DSM-5, among which ASPD, is associated with several drawbacks (Widiger and Samuel, 2005; Rodriguez-Seijas et al., 2019). The categorical way in which personality disorders are classified results in high comorbidity between diagnoses (Widiger and Samuel, 2005). The classifications contain arbitrary thresholds and draw a line between normal and abnormal functioning (Widiger and Samuel, 2005; Rodriguez-Seijas et al., 2019). This may result in failing to classify psychopathology, despite significant suffering. Broad behavioral criteria also lead to heterogeneity within classifications (Rodriguez-Seijas et al., 2019). Several models are proposed in response to these drawbacks. In the developmental process of the DSM-5, discussions about the categorical conceptualization of personality disorders led to a dimensional model to meet the variation in characteristics of individuals. This model consists of a dimensional description of personality pathology. The classification of personality disorders is based on seven criteria (A-G), where criteria A and B are the core criteria. Criteria A describes impairments in self-functioning (identity and self-direction) and interpersonal functioning (empathy and intimacy) on a severity level. Criteria B specifies pathological personality traits based on the Big Five Personality traits (negative affectivity, detachment, antagonism, disinhibition, and psychoticism). These traits describe personality in a dimensional way. Criteria C-G include descriptions of the inflexibility and stability of traits, and differential diagnostics. This dimensional way of describing personality dysfunction meets the heterogeneity and comorbidity of psychopathology (Skodol et al., 2015). Another advantage of the model is its clinical utility in terms of clinical decision-making (Waugh et al., 2017). The model has the potential to improve case conceptualizations and treatment planning (Rodriguez-Seijas et al., 2019). However, the dimensional model turned out to be too complex for clinical practice in the developmental phase of the DSM-5 (Krueger and Markon, 2014), meaning that in line with earlier versions of the DSM, ASPD sustained to be defined with mainly behavioral criteria. Several authors mentioned that the behavioral criteria used for classifying ASPD are just a limited representation of the complexity of ASPD and its underlying etiologies (Brazil et al., 2018; Jurjako et al., 2020; Münch et al., 2020; Rokop et al., 2021).

A model that does have a dimensional character to describe individual differences and addresses these limitations of the DSM, is the Hierarchical Taxonomy of Psychopathology (HITOP; Kotov et al., 2017). Similar to the proposed dimensional model of the DSM-5, the HITOP model describes maladaptive traits on a continuum, which avoids stigmatization by refraining from categorizing individuals in abnormal and normal functioning. The HITOP model covers several spectra, such as internalizing and externalizing spectra. ASB is described in two externalizing spectra. These spectra are distinguished by their underlying tendencies. The disinhibited externalizing spectrum includes impulsive tendencies, which can be associated with for example substance abuse and theft (Kotov et al., 2017). The antagonistic externalizing spectrum includes hostile tendencies accompanied by the violation of the rights of others, which can result in, for example, aggressive behavioral patterns (Kotov et al., 2017). The HITOP model allows comorbidity among mental disorders by describing traits on dimensional spectra. Furthermore, this model also adopts the heterogeneity of diagnoses. Finally, it accepts in line with earlier findings that ASB can be associated with multiple mental disorders (Beauchaine and Hinshaw, 2014).

Where the HITOP model addresses the limitations of the DSM-5 concerning the lack of dimensionality, others have tried to overcome limitations regarding the use of observable behavior in classifying ASPD. Brazil et al. (2018) noticed that the focus on observable behavior alone obscures the complexity of antisocial behavioral problems. They suggest a new approach in which they examine the underlying bio cognitive mechanisms to divide antisocial individuals into subgroups. These subgroups can then be matched to specific therapeutic interventions. When, for example, individuals experience deficits in directing attention to contextual cues, therapeutic interventions can be used to train these specific attention problems to become more aware of those contextual cues and to adopt appropriate behavior and inhibit ASB. Brazil et al. (2018) made a major step forward in presenting a clinically relevant model by adopting the heterogeneity of ASPD and proposing different pathways to ASB. In line with Brazil et al. (2018) we endorse the concerns about the classification of ASPD in the DSM and the scarcity of evidence-based treatments.

Also, Frick and Dickens (2006) proposed a model for explaining ASB, specifically in youth. They provide more insight into early developmental factors of ASB in childhood and adolescence to improve diagnostic classification and treatment indications. They proposed a model in which three dimensions identify antisocial youth: callous-unemotionality, narcissism, and impulsivity. They identified the dimension of callous-unemotionality as the most critical factor for distinguishing severity and persistence of antisocial outcomes in antisocial youth (Frick et al., 2014). By illustrating which factors may influence conduct problems in high- and low-callous-unemotional youth, they provide indications for tailoring interventions to these subgroups. Their way of thinking is inspiring for improving the understanding of ASB in the adult population.

The dimensional character of the HITOP model, the developmental model of Frick and Dickens (2006), and the bio cognitive approach of Brazil et al. (2018) contribute to a better understanding of ASB. These approaches also provide indications for applying therapeutic interventions to specific patient groups. An important contribution of the HITOP model is that therapeutic interventions can target transdiagnostic phenomena that may underlie multiple disorders. However, current research is primarily focused on the efficacy of therapeutic interventions for internalizing psychopathology of the HITOP model (Hopwood et al., 2020). More attention is needed for the externalizing spectra including antisocial behavioral problems.

Moreover, in clinical practice progression is needed to support therapists in making substantiated choices about which intervention can be used for a specific patient exhibiting ASB. Uncertainty about treatment choices has a negative effect on clinicians’ motivation to treat clients with ASB (Van Dam et al., 2022). The model proposed by Brazil et al. (2018) makes an important contribution to the demand for a clinically relevant model. However, this model is primarily focused on the psychopathic subgroup. ASB, however, is common in a broad group of mental health patients (Beauchaine and Hinshaw, 2014; Van Dam and Rijckmans, 2020) and there is only a limited overlap between ASPD and psychopathy (Miller et al., 2001; Venables et al., 2014). Frick et al. (2014), on the other hand, distinguish between youth with high and low callous-unemotional traits. They provide important implications for diagnostic classification for both subgroups. However, their model is based on conduct problems in youth and their research is primarily focused on implications for treatment in youth with elevated callous-unemotional traits. Moreover, it is uncertain to what extent models based on research among juveniles are fully applicable to the adult population. For example, Moffitt (2018) found that a substantial proportion of juveniles with ASB no longer exhibit behavioral problems in adulthood. These antisocial behavioral problems can be considered as part of a more or less normal transition phase to adulthood in male adolescents. A similar pattern has been observed in impulsivity. In a substantial proportion of juveniles, a high degree of impulsivity decreases to normal proportions in adulthood (Hammond et al., 2011). The juvenile and adult populations could therefore have similarities as well as differences in developmental ways to ASB. Therefore, we would like to extend the work of Brazil et al. (2018) and Frick et al. (2014) by proposing a broad spectrum of underlying dynamics of ASB in adults in a new conceptual framework, which covers ASB in the entire mental health population.

This framework may elucidate the controversy in literature regarding contradicting findings in experimental research (e.g., different outcomes in emotion recognition, personality traits and mentalizing functioning) and the limited therapy effects in this population. We intend to provide directions for focusing therapeutic interventions at specific dynamics that may underlie ASB. We will first describe ASB as a dimensional construct related to prosocial behavior. We will do so by focusing on the factors *trust* and *reciprocity* as underlying constructs of (anti)social behavior in line with the reciprocal altruism theory (Trivers, 1971). We will explain these constructs in more detail below and discuss how these factors influence the development and maintenance of ASB. Based on these factors, we will present a new conceptual framework containing different pathways to ASB. Finally, we will suggest how this framework can explain contradictory findings in experimental research and how it may contribute to the improvement of diagnostics and psychological interventions in the antisocial population.

## Antisocial behavior on a continuum with prosocial behavior

One way to get a better conceptual understanding of the dimensionality and heterogeneity of ASB may be to start with a better understanding of why most people behave prosocially and are willing to cooperate and empathize with each other. Prosocial behavior includes ‘concern for others’ wellbeing, empathic (…) and moral focused behaviors, joy at relieving suffering, distress at causing suffering, and capacities for remorse and guilt’ (Gilbert and Basran, 2019; p. 3). ASB can be conceptualized on a continuum with prosocial behavior, described as a diminished ability or willingness to act prosocial.

The question arises why some people tend to act more prosocial than others. An influential theory based on the evolution theory, the reciprocal altruism theory, explains why most people show prosocial behavior (Trivers, 1971). Reciprocal altruism is conceptualized as helping another person, while accepting that this act is incurring some costs. It can be beneficial to make these costs when there is a chance that in the future the other person will be helpful in return. From an evolutionary perspective, this behavior is advantageous for survival. It promotes equality and a peaceful conflict resolution (Gilbert and Basran, 2019). This brings up the question why some people act more antisocial despite these advantages in social life?

**Figure 1:**
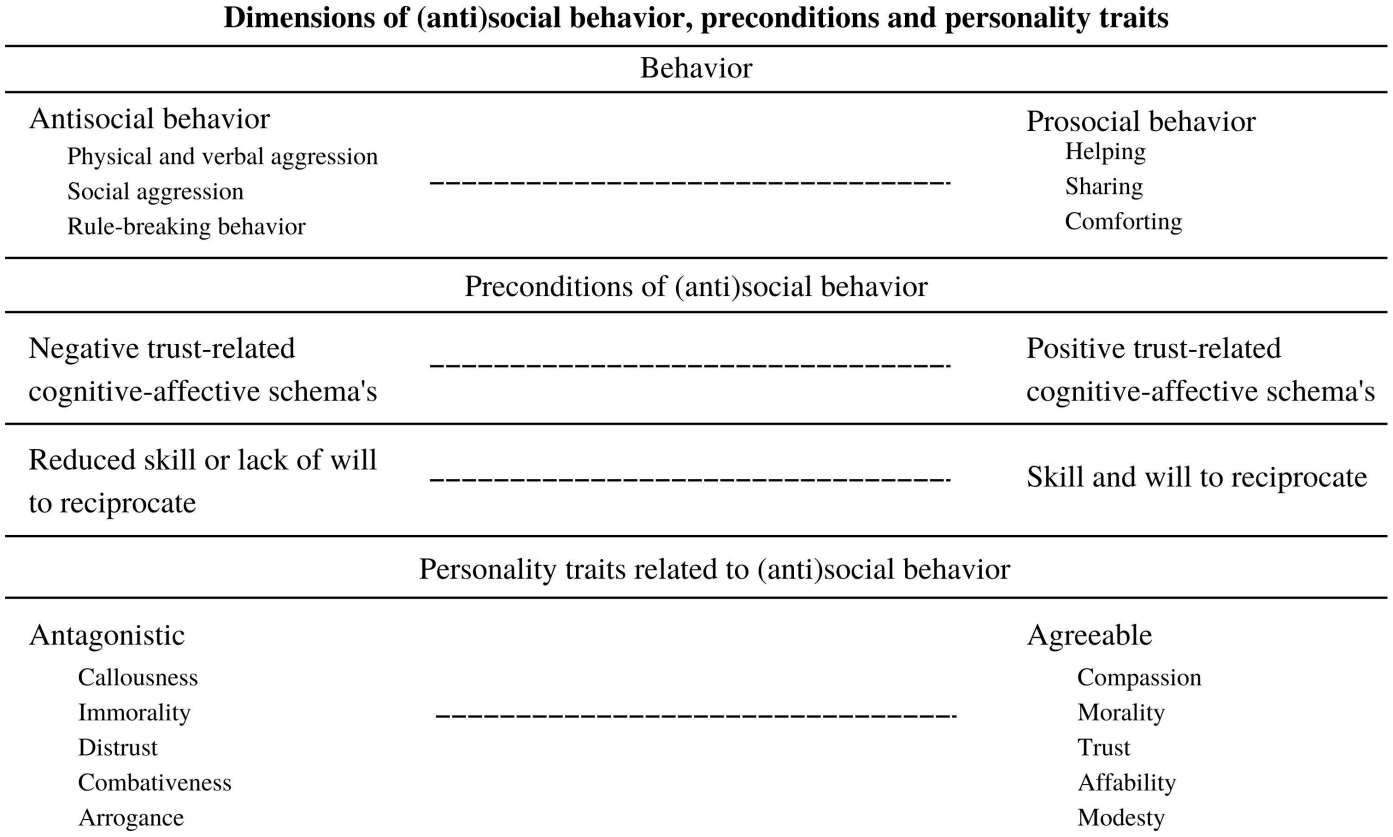
Presents a dimensional model of antisocial behavior in relation to prosocial behavior. Trust and reciprocity can be seen as central processes (i.e., preconditions) in understanding social behavior. For exhibiting prosocial behavior, people need a basic sense of trust in the benign intentions of others. Furthermore, they need skills to show prosocial behavior and the will to apply prosocial behavior. Also, personality traits seem to follow the antisocial-prosocial continuum. Specifically, prosocial behavior is associated with Agreeableness and antisocial behavior with Antagonism.

Figure 1 displays the continuum between prosocial and antisocial behavior. ASB is described as acts of physical and verbal aggression, social aggression and rule-breaking behavior, where prosocial behavior is, in line with the definition of Gilbert and Basran (2019), characterized by helping, sharing and comforting. Prosocial interaction is more likely when the preconditions ‘trust and reciprocity’ are met. These preconditions are explained in further detail in the following paragraph. Besides these preconditions, personality traits are found to be related to (anti)social behavior. Specifically, antagonistic personality traits are associated with antisocial behavioral problems (Decuyper et al., 2009; Lynam and Miller, 2019). The role of personality traits will be later discussed in more detail.

## Preconditions of (anti)social behavior

Central processes for understanding variations in social behavior are trust and reciprocity (Berg et al., 1995; Ostrom and Walker, 2003; Balliet and Van Lange, 2013). Research shows that feelings of (mis)trust about the intentions of others play a central role in how we interact (Van Doesum et al., 2013; Fett et al., 2014; Engelmann et al., 2019; Fareri, 2019) and can therefore be seen as a precondition of social behavior (see Figure 1). Feelings of mistrust are represented in negative trust-related cognitive-affect schemas (Young et al., 2003), resulting in expecting malign behavior from others in social interaction (Balliet and Van Lange, 2013). Mistrust affects behavior, for example in approaching or avoiding others and behaving more or less prosocially (Fett et al., 2014; Fareri, 2019). As can be seen in Figure 1, behavior is expected to be more antisocial-orientated when negative trust-related schemas are present. Later, we will discuss this in more detail. Apart from trust, individuals also need skills to reciprocate with others. For example, abilities to take another’s perspective motivates prosocial interaction (Van Doesum et al., 2013). Besides being able to act prosocial, individuals must also be motivated to do so (Van Doesum et al., 2013). In the antisocial population, some individuals seem to have reduced skills to reciprocate (Newbury-Helps et al., 2017), others seem to show a lack of will to act prosocial or purposely violate rights of others (Woodworth and Porter, 2002; Flight and Forth, 2007). Reduced skills or will to reciprocate are expected to contribute to more ASB, as presented in Figure 1. Trusting others and being able and motivated to reciprocate with them, can thus be seen as essential conditions to exhibit prosocial behavior (Berg et al., 1995; Ostrom and Walker, 2003; Nowak, 2006; Balliet and Van Lange, 2013; Ibáñez et al., 2016; Engelmann et al., 2019; Fareri, 2019). Prosocial behavior may then be seen as the ‘ability and willingness’ to ‘trust and reciprocate’. As presented in Figure 1, exhibited behavior will expected to be closer to the antisocial end of the continuum when one or more of these conditions are challenged. It is worthwhile to discover which factors influence trust and reciprocity in relation to ASB.

## Conceptual framework of antisocial behavior

**Figure 2:**
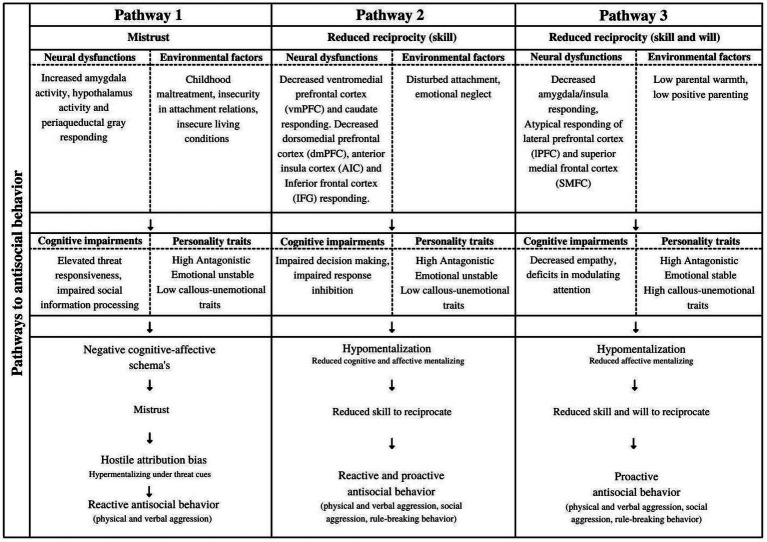
Presents the hypothesized conceptual framework of antisocial behavior based on ‘trust’ and ‘reciprocity’ as preconditions of the prosocial-antisocial dimension. Neural dysfunctions and environmental factors are presented as influencing factors in cognitive impairments and personality traits, which are associated with specific pathways of antisocial behavior. Furthermore, each pathway contains central concepts that may explain developmental ways to antisocial behavior. This framework will be further explained in this paper.

In Figure 2, we present our hypothesized conceptual framework of ASB based on trust and reciprocity (skill and will). This framework covers central underlying factors of ASB based on previous research. This framework is not intended to be exhaustive and includes factors that have clinical relevance. Three hypothesized pathways are presented. Each pathway comprises a developmental pathway to ASB. It is important to mention that the proposed pathways are not mutually exclusive. Individuals may exhibit characteristics that may fit more than one pathway. Therefore, the proposed framework can be seen as a dimensional model, where individuals can present specific dynamics that, to a greater or lesser extent, match one or more developmental ways to ASB. The interaction of these dynamics may even reinforce each other. In the following part of this paper, we will first describe why some individuals are more susceptible for externalizing psychopathology instead of internalizing psychopathology by outlining the role of personality traits in ASB. Next, each element of the proposed pathways will be carefully explained.

## Personality traits: Role in externalizing psychopathology and heterogeneity of antisocial behavior

Internalizing psychopathology is common in mental healthcare settings. However, some individuals seem to be more susceptible to externalizing psychopathology, such as ASB. How can these differences in expressions of psychopathology be explained? Several researchers mentioned personality traits as a possible transdiagnostic factor for explaining externalizing psychopathology (Lynam and Miller, 2019; Vize et al., 2019). Thereby, personality traits are also suggested to contribute to the heterogeneity of the antisocial population (Miller and Lynam, 2006; Vize et al., 2019), which may explain differences is antisocial expressions, severeness and frequency of ASB within the antisocial population.

The five-factor model of personality (FFM; Costa and McCrae, 1992) is frequently used to investigate relations of personality traits with ASB. The FFM contains five personality factors: Agreeableness, Conscientiousness, Neuroticism, Openness, and Extraversion, and describes personality in a dimensional way. Miller and Lynam (2001) reported that Agreeableness, Conscientiousness, and Neuroticism have the most explanatory value in relation to ASB. Especially low Agreeableness, also known as Antagonism, is frequently found as the most robust contributor to ASB (Decuyper et al., 2009; Lynam and Miller, 2019; Vize et al., 2019). The level of Agreeableness or Antagonism reflects individual differences in the orientation toward others. A more antagonistic personality can be described as a general tendency to be hostile, suspicious, and less empathic and cooperative in relation with others. Individuals who have more antagonistic traits are more likely to be aggressive than persons who are more agreeable (Vize et al., 2019), which may explain why some people show externalizing instead of internalizing psychopathology. Furthermore, the level of Antagonism within the antisocial population also seems to variate (Decuyper et al., 2009). For example, people with a high level of psychopathic traits are characterized by a significantly higher level of Antagonism (Hicklin and Widiger, 2005; Ruiz et al., 2008; Decuyper et al., 2009). Decuyper et al. (2009) found non-overlapping confidence intervals in levels of Antagonism in ASPD and psychopathy. This possibly explains differences in frequency and severeness of ASB within the antisocial population and underlines the heterogeneity of the antisocial population.

Another personality factor that has explanatory value in the heterogeneity of the antisocial population and distinguishes between different expressions of ASB is Neuroticism. Vize et al. (2019) found that Neuroticism was more related to reactive aggression than proactive aggression. Individuals high on Neuroticism are more emotional unstable and experience more negative emotions such as anxiety, anger, and depression. Within the antisocial population, some individuals seem more emotionally unstable than others (Del Gaizo and Falkenbach, 2008). These characteristics make it more likely to react with aggression due to their susceptibility for emotional dysregulation (Miller and Lynam, 2006; Sargeant et al., 2011; Miller et al., 2012; Luyten and Fonagy, 2015; Reardon et al., 2018). High Neuroticism can also influence the emotional sensitivity for trauma complaints (Ogle et al., 2017) and may influence the emotional reaction on negative life events (Sun et al., 2016), which can trigger aggressive behavior (Sun et al., 2016).

Antisociality is also related to low Conscientiousness. This is expressed by a high level of impulsiveness and failing to oversee consequences of behavior (Miller et al., 2012). A high level of impulsiveness enhances the likelihood to exhibit ASB (Miller et al., 2012). However, Gray et al. (2019) found that the interpersonal traits of psychopathy are related to low impulsivity, which is in line with the tendency of ‘emotional stable psychopaths’ to plan their behavior carefully and being goal-directed (Hicks et al., 2004).

Personality traits serve as an important factor in differentiating externalizing and internalizing psychopathology. Furthermore, they explain a part of the heterogeneity within the antisocial population (Decuyper et al., 2009; Lynam and Miller, 2019). However, questions about developmental pathways to ASB still remain. To give more insight in pathways that may lead to ASB, we will now explain the central concepts of these pathways, based on ‘trust’, and ‘reciprocity’ as presented in Figure 2.

## Trust

Trust is defined as ‘generalized beliefs and attitudes about the degree to which other people are likely to be reliable, cooperative, or helpful, independent of the specific context or situation in which an interaction with them might take place’ (Simpson, 2007; p. 588). Therefore trust is an essential underlying factor of prosocial interaction, in which people expect benign behavior from others and interact in a cooperative way (Thielmann and Hilbig, 2015). Mistrust as the counterpart of trust can be described as the expectation of malign behavior from others (Balliet and Van Lange, 2013) and can therefore result in negative attitudes and behavior toward others (Thielmann and Hilbig, 2015). When people mistrust others, they may be unable as well as unwilling to act prosocial. Trust can be linked to personality traits (Costa and McCrae, 1992) and is shaped by early childhood experiences (Bowlby, 1969).

Children raised in a predictable childhood environment are more likely to develop a basic sense of trust in others compared to children raised in unstable childhood environments (Wu et al., 2017). The latter seem to be more suspicious about the intentions of others due to negative experiences with trust (Wu et al., 2017), which can be seen as a mechanism of self-protection to avoid being hurt or taken advantage of. This is in line with the attachment theories of Erikson (1963) and Bowlby (1969), which state that children learn in relation to their primary caregivers whether their needs will be met and whether they can rely on the responsiveness of significant others. When there is a stable pattern of responsiveness, children develop positive working models of trust, also known as ‘schemas’. Schemas are mental representations containing cognitive and emotional patterns about the person’s self, others, and the world (Young et al., 2003). Positive experiences in childhood can be seen as supportive to the development of a basic sense of trust (Simpson, 2007). Negative trust experiences (i.e., trauma exposure) can adversely influence the psychological development of children, resulting in negative cognitions and emotions about the person self, others and the world (Young et al., 2003; Simpson, 2007; Bateman and Fonagy, 2012; Thielmann and Hilbig, 2015). These acquired schemas influence future social interactions and explain differences in human behavior (Bateman and Fonagy, 2012; Thielmann and Hilbig, 2015).

Traumatic experiences in childhood can be seen as negative trust experiences that may result in feelings of mistrust (Bateman and Fonagy, 2012). Trauma exposure is frequently found in antisocial populations (Lobbestael et al., 2005; Chen et al., 2012; Stimmel et al., 2014). Prevalence rates of 86–94% of trauma exposure have been found in offenders (Abram et al., 2004; Rosenberg et al., 2014; Stimmel et al., 2014). Specifically, childhood maltreatment and feelings of insecurity in attachment relations can increase the likelihood of developing feelings of mistrust toward others (Bateman and Fonagy, 2012; Zhu et al., 2020). Additionally, insecure living conditions in adulthood also may lead to dysfunctional schemas (Ripley et al., 2019). Therefore, a part of the antisocial population with traumatic experiences has a potential risk for dysfunctional schemas about self, others and the world. Moreover, negative trust experiences may continue across lifetime in antisocial individuals. ASPD, for example, is characterized by a pattern of interpersonal conflicts and violence (American Psychiatric Association, 2013), which is accompanied by unsafe living conditions throughout the lifespan. Feelings of unsafety may confirm dysfunctional schemas about mistrust learned in childhood. It is therefore not surprising that feelings of mistrust are associated with a part of the antisocial population (Klein Tuente et al., 2019).

The relationship between feelings of mistrust and ASB becomes more clear when exploring information processing in social interaction in more detail. Bateman and Fonagy (2012) explain how negative cognitions in people can shape their relationships and influence how they interact. Challenged epistemic trust can lead to a biased perception of social reality when the interpretation of others’ intentions is based on maladaptive cognitions. Epistemic trust, as introduced by Bateman and Fonagy (2012), includes openness to social information and adopting this information as personally relevant and generalizable. When epistemic trust is challenged, a more biased perception of social reality is more likely (Bateman and Fonagy, 2012; Fonagy et al., 2015). In social interaction, people have to be able to estimate others’ mental states correctly, to make adequate decisions about approaching or avoiding the other person (Fareri, 2019). The social information processing (SIP) model (Dodge and Crick, 1990) delineates that encoding and interpreting cues is an essential step in social interaction to make decisions about responding. However, the process of encoding and interpreting intentions of others can be susceptible to mistakes. Maladaptive cognitions based on earlier negative trust experiences can enhance the chance of misinterpretations (Bateman and Fonagy, 2012). Traumatized individuals have minimal confidence that others will act benevolently toward them, which may lead to a wrong assessment of others’ intentions (Simpson, 2007; Chen et al., 2012).

## Pathway 1: Trust in relation to antisocial behavior

Negative trust experiences may lead to ASB when others’ intentions are wrongly assessed. Research has noted that aggression is associated with a pattern of misinterpretation of stimuli, especially labelling stimuli as threatening or hostile (Bradshaw and Garbarino, 2006; Chen et al., 2012). This tendency is also known as a hostile attribution bias (HAB), a specific form of hypermentalization (Milich and Dodge, 1984; De Castro et al., 2002; Abate et al., 2017; Smeijers et al., 2017). Trust can thus be seen as essential condition for developing adequate mentalizing capacities (Bateman and Fonagy, 2012). If trust is challenged, hostile attribution biases are more likely. Hostile attribution bias is a well-known phenomenon in the antisocial population (Dodge and Coie, 1987; Schwartz et al., 1998; Aber et al., 2004; Abate et al., 2017; Smeijers et al., 2017; Klein Tuente et al., 2019). Researchers found that aggressive individuals assess neutral and ambiguous faces more frequently as hostile (Burt et al., 2009; Schonenberg and Jusyte, 2014) and that these hostile biases predict reactive aggression (Lobbestael et al., 2013; Richey et al., 2016). It is not unlikely that the frequent presence of hostile attribution biases in the antisocial population can be attributed to prior negative trust experiences. Besides negative trust experiences (i.e., environmental factors) may increase the likelihood of reactive aggression, also cognitive impairments may be of importance. The chance to show reactive aggression can be enhanced by elevated sensitivity for threat (Crowe and Blair, 2008; White et al., 2016). This sensitivity for threat is related to neural dysfunctions. Specifically, associations with increased amygdala and hypothalamus activity and periaqueductal gray responding are found (Blair et al., 2018). However, environmental factors, such as trauma symptoms, may also affect this threat sensitivity (Blair et al., 2018). Furthermore, specific personality traits seem to play an important role in influencing the interaction between above-mentioned underlying factors. High Antagonism may increase the likelihood to show ASB (Miller et al., 2012), but is also related to specific proneness to hostile attribution biases (Miller et al., 2008). High Neuroticism is also found in a part of the antisocial population and covers emotional unstable traits. Emotional instability may influence the emotional reaction to negative life events and then trigger ASB (Sun et al., 2016). Summarizing, we hypothesize, as described in Figure 2, that challenged trust can be seen as a central factor in the development of hostile biases in the antisocial population. Specifically, cognitive dysfunction (threat sensitivity, impaired information processing) and environmental factors (e.g., childhood abuse, insecurity in attachment relations and insecure living conditions) may be underlying the development of hostile biases and may result in reactive ASB, specifically physical and verbal aggression. Social aggression and rule-breaking behavior are less likely to be influenced by mistrust and hostile biases and are therefore not included in pathway 1.

## Reciprocity

How can ASB be explained when people do not experience negative cognitions about trusting others? Reverting to the reciprocal altruism theory for exhibiting prosocial behavior, people also need skills to reciprocate with others and the will to do so (Van Doesum et al., 2013). The ability to take another person’s perspective into account can be seen as an essential condition for being prosocial toward others and to reciprocate with them (Van Doesum et al., 2013; Fett et al., 2014). This skill is also known as mentalizing ability, which requires ‘perceiving and interpreting feelings, thoughts, beliefs and wishes’ (Bateman and Fonagy, 2012) (p. 3). Individuals vary in strengths and competences with regard to the ability to mentalize. Besides the hypermentalizing modes found in relation with mistrust in the antisocial population, also low (i.e., underdeveloped) mentalizing capacities are recurrently found (Bateman et al., 2013; Newbury-Helps et al., 2017; Abi-Habib et al., 2020).

Mentalization is a complex phenomenon and people’s mentalizing skills can be expressed in different areas. Research is often focused on part of these areas, such as emotion recognition, affective resonance or more advanced mentalizing skills such as theory of mind and faux-pas tasks (Dolan and Fullam, 2004; Jones Bartoli et al., 2007; Shamay Tsoory et al., 2010; Newbury-Helps et al., 2017; Gillespie et al., 2018; Velotti et al., 2019; Abi-Habib et al., 2020). Regarding emotion recognition, at least a part of the antisocial population seems to be less able to recognize emotions based on facial expressions. Some researchers reported deficiencies in recognition of negative affect, such as sadness (Blair et al., 2001; Dolan and Fullam, 2006), fear (Blair et al., 2001; Montagne et al., 2005; Marsh and Blair, 2008) and anger (Jones Bartoli et al., 2007). Others found more general impairments in emotion recognition (Chapman et al., 2018). There is also evidence for general poor perspective taking skills in the antisocial population (Fonagy and Levinson, 2004; Moller et al., 2014; Newbury-Helps et al., 2017; Protic et al., 2020) and specific deficiencies in faux pas-tasks (Dolan and Fullam, 2004). Some researchers reported that antisocial individuals have deficiencies primarily in the emotional understanding of the mental state of others, also known as affective mentalizing (Blair et al., 2006; Shamay Tsoory et al., 2010; Bateman and Fonagy, 2012; Bateman et al., 2013). These findings about poor emotion recognition, perspective taking and affective mentalizing contribute to the hypothesis that a part of the antisocial population has underdeveloped mentalizing capacities, also known as hypomentalizing (underattribution of mental states) or in severe form no mentalizing capacities (failure to mentalize).

Different reasons are mentioned in literature as explanation for hypomentalizing modes in the antisocial population. We will briefly highlight some important factors. One explanation for these hypomentalizing modes is insecure attachment in childhood. According to attachment theories (Erikson, 1963; Bowlby, 1969), secure attachment facilitates emotion regulation and the development of adequate mentalizing functioning (Fossati et al., 2009; Bateman and Fonagy, 2012). Disturbed attachment limit opportunities for a child to recognize mental states in his/her caregiver and to internalize these mental states which leads to a delay or inhibition, and thus an underdevelopment, of mentalizing capacities (György and Unoka, 2008). In Fonagy’s theory of mentalization, he proposes that antisocial behavior is brought about by an impaired or underdeveloped ability to represent mental states in oneself and others (Fonagy and Levinson, 2004). Early relational experiences between child and caregivers influence the ability to recognize aggressive impulses and learn alternative ways to express and regulate them (Allen et al., 2008). Disturbed attachment may then negatively affect the development of mentalizing capacities (Fonagy et al., 2007). A lack of attuned parental mirroring of affect (i.e., emotional neglect) can lead to disconnecting oneself from the other and impairments in recognizing and understanding mental states (Bateman and Fonagy, 2012). This leads to the use of anti- or prementalizing modes (i.e., hypomentalization).

Another explanation is that cognitive impairments may play a role (Frick et al., 2014; Blair et al., 2018). Impaired decision-making may enhance the likelihood for ASB. For example, individuals who have more difficulty with predicting (negative) outcomes (e.g., harming others) are less likely to avoid harmful behavior in social interaction (Blair et al., 2018). Decreased ventromedial prefrontal cortex (vmPFC) and caudate responding may related to impairments in decision-making in antisocial populations (Blair et al., 2018). Also impaired response inhibition is found in antisocial individuals (Puiu et al., 2018; Sun et al., 2020). Difficulties in inhibiting impulses during selecting behavioral responses may lead to impulsive ASB (Puiu et al., 2018). Blair et al. (2018) mention decreased dorsomedial prefrontal cortex (dmPFC), anterior insula cortex (AIC) and inferior frontal cortex (IFG) responding as possible underlying neural dysfunctions resulting in impaired response inhibition. Mentalizing requires a careful representation of others perspective to choose non-harming behavior. Both impaired decision-making and response inhibition may influence these capacities. We hypothesize that environmental factors (e.g., disturbed attachment, emotional neglect) and cognitive dysfunctions (e.g., impaired decision-making and response inhibition) may therefore influence the likelihood of hypomentalizing modes.

## Pathway 2: Reciprocity (skill) in relation to antisocial behavior

Reduced skills to act prosocial may lead to ASB. According to the violence inhibition mechanism (VIM; Blair, 2001), seeing distress in others (e.g., sad and fearful faces) can activate autonomic arousal in individuals and subsequently inhibit ongoing destructive behavior. When distress cues are poorly recognized due to hypomentalizing, the VIM may not be activated. This may result in ASB. This is in line with specific personality traits found in the antisocial population. Besides a high level of Antagonism as robust predictor of ASB (Decuyper et al., 2009; Miller et al., 2012), also high levels of impulsivity are found in a part of the population (Miller et al., 2012). High impulsivity can be related to low levels of Conscientiousness (e.g., failing to oversee consequences of behavior) as well as high levels of Neuroticism (impulsive reaction when emotionally dysregulated) and result in ASB (Miller et al., 2012). This is also in line with impaired response inhibition on a cognitive level that may be (partly) underly these impulsive traits. Following the proposed framework in Figure 2, hypomentalization, can then be hypothesized to lead to reactive- and proactive ASB (physical and verbal aggression, social aggression, rule-breaking behavior).

## Pathway 3: Reciprocity (skill and will) in relation to antisocial behavior

In research, some findings indicate that a part of the antisocial population show less impairments in mentalizing abilities. For example, Dolan and Fullam (2004) found that individuals with psychopathic characteristics show only subtle impairments in mentalizing abilities and meta-analytic results indicate that psychopathy is associated with only small deficits in emotion recognition (Wilson et al., 2011). Woodworth and Waschbusch (2008) reported even a better fear recognition in children with high callous-unemotional traits (CU traits). Other researchers reported that individuals with psychopathic characteristics have only specific impairments in affective mentalizing (Dolan and Fullam, 2004; Blair et al., 2006; Shamay Tsoory et al., 2010). Affective mentalizing can be specified as the emotional understanding of others’ feelings and resonate with these mental states. They perceive negative emotions less adverse than others (Blair et al., 2006) and show reduced sensitivity to the emotional value of stimuli (Kosson et al., 2006; Lobbestael et al., 2009; Blair et al., 2018; Marsden et al., 2019) and pain (Blair et al., 2018). This is in line with how Cleckley (1988) described psychopathy. He emphasized differences in the expressed and experienced values of emotions in psychopaths.

We hypothesize that this population with specific mentalizing impairments is characterized by high CU traits. CU traits refer to the affective dimensions of psychopathy, and therefore presents a just small group of psychopathic individuals. CU traits include the lack of empathy and guilt, and shallow or deficient affect. Researchers found that CU traits share mostly the same genetic underpinnings with Big Five personality traits and are related in several ways (Mann et al., 2015). However, CU traits reflect a specific dimensional combination of these personality traits. Associations with Antagonism are consistently reported (Roose et al., 2012; Frick and Ray, 2015). Openness seems also be negatively related to CU traits (Frick and Ray, 2015). Some researchers mentioned a negative association with the facet anxiety of Neuroticism, whereby the facet hostility was positively associated with CU traits (Roose et al., 2012; Frick et al., 2014). CU traits can then be seen as an informative description of a specific group of individuals characterized by low empathy, emotional insensitivity, and fearless traits.

The impairments in emotional functioning in this small antisocial population with high CU traits may partly be due to neurobiological predisposition (Frick et al., 2014). Sebastian et al. (2012) demonstrated that hyporeactivity of the amygdala and anterior insula was related to impairments in affective theory of mind in children with conduct problems. Blair et al. (2018) reported that reduced amygdala functioning is an underlying neural dysfunction of impairments in affective theory of mind and deficiencies in the processing of distress cues and pain. These dysfunctions may lead to reduced empathy in line with the CU characteristics. Another explanation for impaired emotional functioning is an ‘attention bottleneck’ (Baskin-Sommers and Brazil, 2022). When social information is presented within the attention or goal-direction of these antisocial individuals, they may recognize others’ emotions adequately and make inferences about them. However, when information (e.g., affective stimuli/emotions) is presented as ‘secondary’ information or in more complex situations, these individuals have difficulties in recognizing and making inferences about others’ thoughts and emotions (Drayton et al., 2018; Brennan and Baskin-Sommers, 2021; Baskin-Sommers and Brazil, 2022). The latter can lead to their goal-directed ASB when they ignore contextual cues, such as adverse emotions in others. Also, environmental factors may play a role in low empathic and -emotional sensitivity of this subgroup. For example, low parental warmth and low positive parenting are risk factors for the development of CU traits (Hyde et al., 2016; Muratori et al., 2016; Waller et al., 2018). Although a gene-environmental interaction is expected, this small group seems to have a significant heritable pathway (Frick et al., 2014; Hyde et al., 2016).

Returning to the reciprocal altruism theory, people need the skill to act reciprocal but also the will to do so. Research indicates that people with high CU traits do not show overall mentalizing impairments (Wilson et al., 2011; Chang et al., 2021). There is some evidence that genetic etiologies underlie these high CU traits (Moore et al., 2019). This subpopulation commit more ASB than non-psychopathic individuals (Chang et al., 2021) and ASB is more proactive, which can be seen as purposeful and motivated by a specific goal (Cima and Raine, 2009; Gillespie et al., 2018). This purposeful use of violence can possibly be ascribed to specific impairments in mentalizing, which may primarily find its roots in decreased empathy and impairments in attention (i.e., attention bottleneck). A lack of awareness about missed social information and their self-centeredness for achieving their own goal (reduced ‘will’ to act prosocial) may lead to a pattern of callously harming and manipulating others which may be hypothesized as a third pathway to proactive ASB (physical and verbal aggression, social aggression, rule-breaking behavior).

## Overview of pathways to antisocial behavior

We have provided an overview of different dynamics that may underlie ASB. These dynamics form the basis for our proposed conceptual framework, which is presented in Figure 2. We hypothesize that the first pathway is characterized by traumatic experiences (childhood maltreatment, insecurity in attachment, insecure living conditions) and elevated threat responsiveness, which leads to negative cognitive-affective schemas about the person self, others and the world (e.g., mistrust). These maladaptive beliefs affect social information processing resulting in a hostile bias (specific form of hypermentalization). By attributing hostile intentions to others, a person experiences situations of potential threat more often. To prevent oneself of being hurt by others, reactive aggression is a likely reaction. The second pathway describes how cognitive dysfunction (e.g., impaired decision making and response inhibition) and environmental factors (e.g., disturbed attachment, emotional neglect) can lead to hypomentalizing modes. When people, for example, have not been supported in the development of adequate mentalizing in childhood, mentalizing capacities can be negatively affected, which may result in hypomentalization. Hypomentalization may also be driven by emotional instability or vulnerability for stress. Regarding a part of the antisocial individuals seems to experience negative emotions more frequently and intense, low mentalizing modes (e.g., reduced perspective taking) are expected (Bateman and Fonagy, 2008; Bateman et al., 2013). We hypothesize that hypomentalization in combination with emotional instability can lead to reactive and proactive ASB. The third pathway describes individuals who are characterized by relatively intact mentalizing functioning. However, mentalizing functioning can be affected by cognitive impairments, such as decreased empathy or an attention bottleneck, resulting in low affective mentalizing abilities. The inhibiting function of experiencing arousal or stress in others which may prevent for ASB, may then be reduced. We hypothesize that these specific low affective mentalizing abilities are mainly found in individuals with high CU traits. This small group of individuals seems more stable emotionally, which may reinforce their goal-directed and manipulative behavior. We hypothesize that this small group will primarily be associated with proactive ASB. The distinction between pathway 2 and 3 as outlined above is also in line with the proposed pathways of Frick and Dickens (2006) for conduct problems in youth, where high callous-unemotionality is linked to more severe and stable ASB and fearless traits (pathway 3 in the current study) and lower CU traits to more impulsive behavior and emotional dysregulation (pathway 2 in the current study).

## The heterogeneity of antisocial behavior

Our framework adopts ASB as a transdiagnostic and heterogeneous construct through which earlier contradicting findings in experimental research can be explained. Although the antisocial manifestations in the antisocial population are often expressed in similar ways, we suggest that the underlying dynamics are quite different. For example, where some antisocial individuals experienced a history of traumatic events and (mis)attribute hostile intentions to others, others show a more general hypomentalizing tendency in which they have difficulty in recognizing their own and others’ mental states. We assume that this may have resulted in varying (and contradicting) outcomes in the antisocial population on cognitive tasks (Dawel et al., 2012; Chapman et al., 2018; Gillespie et al., 2022). Furthermore, the heterogeneity in the antisocial population may also clarify why several treatments showed limited effect. Treatments may not have been targeted to the specific underlying dynamics in patients, which may have suggested that treatments are ineffective or that antisocial individuals are untreatable. However, instead of labelling this population as untreatable, a thorough understanding of the underlying dynamics can provide valuable information for adapting treatments to the special needs of these individuals. This is also important because research indicates that specific knowledge about applying therapeutic interventions to ASPD patients enhances the motivation of clinicians to work with this population (Van Dam et al., 2022).

## Implications for clinical practice

**Figure 3:**
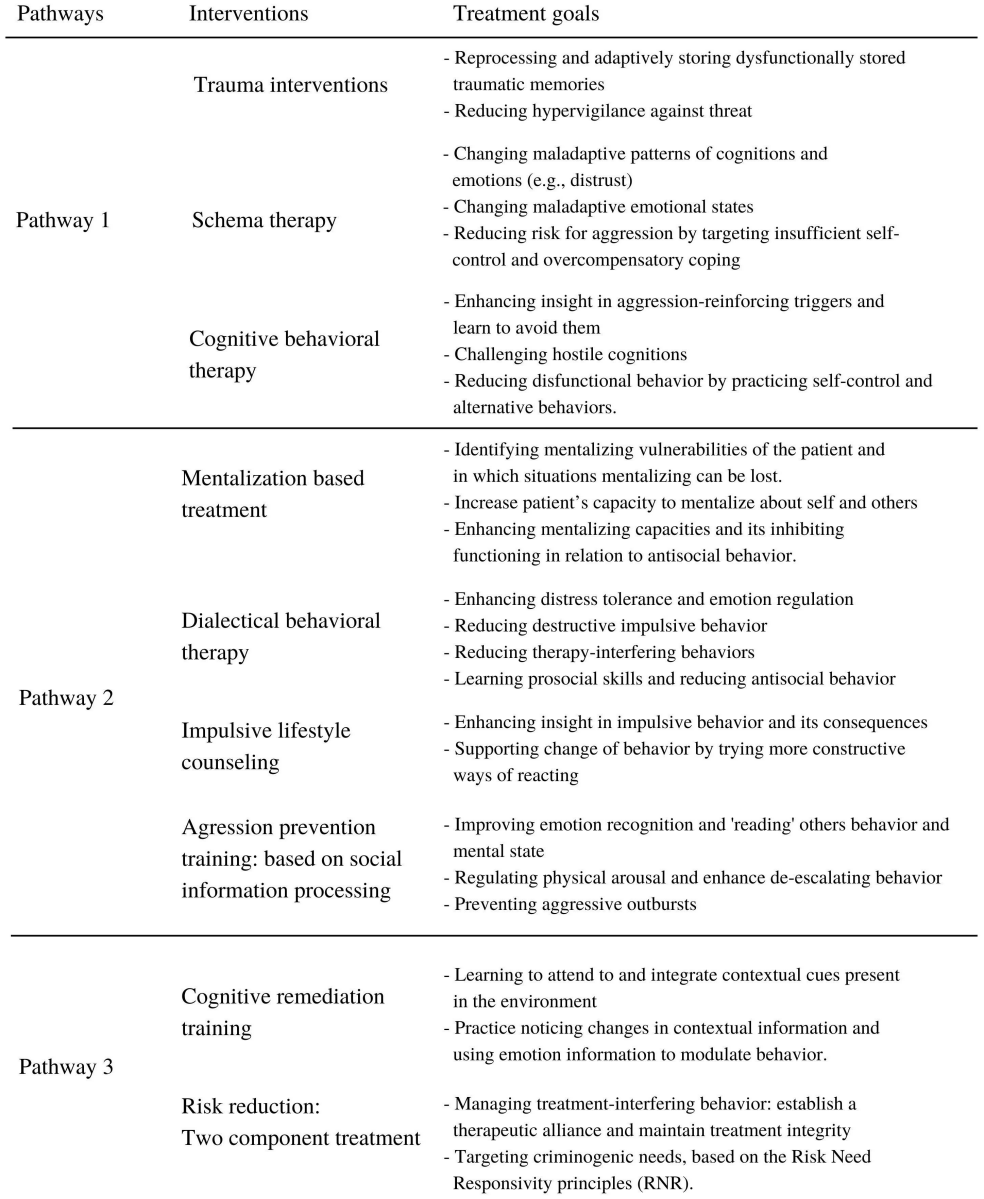
Presents indications for interventions in the antisocial population based on the proposed conceptual framework (Figure 2). These interventions target different dynamics that may underlie antisocial behavior.

The proposed framework is intended to serve as a clinically relevant model that provides directions for improving diagnostics and matching treatments to the underlying dynamics in the antisocial population. Figure 3 presents an overview of therapeutic interventions linked to these different dynamics. When a history of trauma has resulted in feelings of mistrust and hostile attribution biases (pathway 1), we propose that these patients could benefit from treatments targeting trauma and/or negative cognitions about (trusting) others. Research shows promising results regarding trauma-based treatments, such as Eye Movement Desensitisation and Reprocessing (EMDR), in antisocial samples. EMDR-therapy resulted in reduced problem behavior in youth- (Soberman et al., 2002; Farkas et al., 2010; McMullen et al., 2013) and adult samples (Ricci et al., 2006; Wright and Russell, 2013). EMDR also enhanced the amount of empathy for victims (Ricci et al., 2006; Van Tilburg, 2020). Schema therapy, which targets negative cognitions about self, others and the world, presented strong reductions on personality disorder-symptoms and self-control (Bernstein et al., 2021). Schema therapy showed even significant improvements in psychopathic characteristics, negative cognitions and risk-related behaviors (Chakhssi et al., 2014). It also enhanced control of behavior in emotional situations and it reduced anger (Smith, 2011). Several interventions based on cognitive behavioral therapy (CBT) have also been developed to target aggression (Dorrepaal et al., 2008; Van Dam et al., 2012). Results indicate that CBT can contribute to reduce hostility and psychopathology (Serie et al., 2015).

Other individuals may tend to hypomentalize and have more difficulties with response inhibition and decision-making (pathway 2). To improve mentalizing abilities, Mentalization Based Treatment (MBT) may be helpful (Bateman and Fonagy, 2012, 2019). MBT is aimed at improving the recognition of one’s own and others’ mental states (Bateman and Fonagy, 2012). Research showed promising results for individuals with ASPD regarding anger and hostility reduction and impulse control (Bateman et al., 2016). It also enhanced patients mentalizing capacities and empathy for others (Ware et al., 2016). For reducing impulsivity in individuals with ASB, Impulsive Lifestyle Counselling is a promising intervention. Researchers found reduced substance abuse and treatment drop-out in individuals with an antisocial personality disorder (Thylstrup et al., 2015, 2017). Another intervention that targets emotional instability and impulsivity is Dialectical Behavior Therapy (DBT). Research findings indicate that DBT is helpful in reducing verbal and physical aggression and criminal offending (Burt and Donnellan, 2009; Wetterborg et al., 2020). An aggression prevention training based on improving cognitive-emotional skills, specifically on emotion recognition and emotion regulation (VRAPT; Klein Tuente et al., 2020), did not reduce overall aggressive behavior in a general forensic inpatient sample, but did temporarily reduce hostility, anger control and impulsiveness. However, this intervention might be more effective when offered to patients with specific impairments in these cognitive-emotional skills.

For antisocial individuals with high callous-unemotional traits, interventions that target underlying impairments are extremely scarce. However, results in the study of Baskin-Sommers et al. (2015) provide foundation for cautious optimism that specific cognitive interventions may be helpful to reduce underlying information processing impairments in psychopaths. Individuals high on interpersonal and affective characteristics of psychopathy followed a cognitive remediation training in which ‘attention-to-context’ was trained. After the training, these individuals were more responsive to affective and nonaffective information and were more able to divide their attention to goal-specific stimuli and the context. Additionally, interventions targeting risk reduction also show positive results. Wong and Gordon (2013) described a two-component treatment for psychopathic individuals to manage antisocial behavior. The first component is to manage treatment-interfering behavior originating from affective and interpersonal impairments in this population. The second component is targeting their criminogenic needs. This intervention resulted in reduced recidivism in psychopathic individuals (Wong et al., 2012). These results confirm that targeting underlying dynamics or taking these dynamics into account when treating this population, provides hopeful results for the antisocial population.

## Conclusion

We proposed a conceptual framework with regard to the underlying dynamics of ASB, which explains contradictory research outcomes and limited therapeutic effects. Most important, we tried to create a fundament for improved diagnostics of the antisocial individual that may serve as a guide for matching therapeutic interventions to individuals’ needs.

However, we acknowledge that further research is necessary to empirically test the proposed framework and its related constructs in relation to different forms of ASB. Main objectives for further research are to investigate how the above-mentioned predictors in each pathway relate to ASB, whether the proposed pathways can be confirmed in experimental research, and which population sizes correspond to the pathways. Data collection has already started in mental health care centers in the Netherlands, where patients with antisocial behavioral problems (e.g., physical aggression, social aggression and/or rule-breaking behavior) are included. Multiple instruments are administered to get more insight in predictors of ASB. Study results may contribute to new approaches of diagnostics and treatment programs for ASB.

## Data availability statement

The original contributions presented in the study are included in the article/supplementary material, further inquiries can be directed to the corresponding author.

## Author contributions

BW and AD did the main work in conceptualizing the framework. BW wrote the first draft of the manuscript. MR aided in conceptualizing. AD, MR, and JV contributed to revising and editing of the manuscript. All authors contributed to the article and approved the submitted version.

## Conflict of interest

The authors declare that the research was conducted in the absence of any commercial or financial relationships that could be construed as a potential conflict of interest.

## Publisher’s note

All claims expressed in this article are solely those of the authors and do not necessarily represent those of their affiliated organizations, or those of the publisher, the editors and the reviewers. Any product that may be evaluated in this article, or claim that may be made by its manufacturer, is not guaranteed or endorsed by the publisher.

## References

[ref1] Abate A. Marshall K. Sharp C. Venta A. (2017). Trauma and aggression: investigating the mediating role of mentalizing in female and male inpatient adolescents. Child Psychiatry Hum. Dev. 48, 881–890. doi: 10.1007/s10578-017-0711-6 28176177

[ref2] Aber J. L. Gershoff E. T. Ware A. Kotler J. A. (2004). Estimating the effects of September 11th and other forms of violence on the mental health and social development of New York City's youth: a matter of context. Appl. Dev. Sci. 8, 111–129. doi: 10.1207/s1532480xads0803_2

[ref3] Abi-Habib R. Wehbe N. Badr K. Tohme P. (2020). Do prisoners mentalize differently? Investigating attachment and reflective functioning in a sample of incarcerated Lebanese men. Int. J. Forensic Ment. Health 19, 183–197. doi: 10.1080/14999013.2019.1684403

[ref4] Abram K. M. Teplin L. A. Charles D. R. Longworth S. L. McClelland G. M. Dulcan M. K. (2004). Posttraumatic stress disorder and trauma in youth in juvenile detention. Arch. Gen. Psychiatry 61, 403–410. doi: 10.1001/archpsyc.61.4.403 15066899 PMC2861915

[ref5] Allen J.G. Fonagy P. Bateman A. (2008). Mentalizing in Clinical Practice. Washington, DC: American Psychiatric Pub.

[ref1530] American Psychiatric Association (2013). Diagnostic and statistical manual of mental disorders. 5th Edn. Washington, DC: Author.

[ref6] Balliet D. Van Lange P. (2013). Trust, conflict, and cooperation: a meta-analysis. Psychol. Bull. 139, 1090–1112. doi: 10.1037/a0030939 23231532

[ref7] Baskin-Sommers A. Brazil I. A. (2022). The importance of an exaggerated attention bottleneck for understanding psychopathy. Trends Cogn. Sci. 26, 325–336. doi: 10.1016/j.tics.2022.01.001, PMID: 35120814

[ref8] Baskin-Sommers A. R. Curtin J. J. Newman J. P. (2015). Altering the cognitive-affective dysfunctions of psychopathic and externalizing offender subtypes with cognitive remediation. Clin. Psychol. Sci. 3, 45–57. doi: 10.1177/2167702614560744, PMID: 25977843 PMC4426343

[ref9] Bateman A. Bolton R. Fonagy P. (2013). Antisocial personality disorder: a mentalizing framework. Focus 11, 178–186. doi: 10.1176/appi.focus.11.2.178

[ref10] Bateman A. Fonagy P. (2008). Comorbid antisocial and borderline personality disorders: mentalization-based treatment. J. Clin. Psychol. 64, 181–194. doi: 10.1002/jclp.20451 18186112

[ref11] Bateman A. Fonagy P. (2012). Handbook of Mentalizing in Mental Health Practice. Washington, DC: American Psychiatric Pub.

[ref12] Bateman A. Fonagy P. (2019). ‘’Mentalization-based treatment for borderline and antisocial personality disorder’’ in *Contemporary psychodynamic psychotherapy: Evolving clinical practice*. eds. D. Kealy and J. S. Ogrodniczuk (Cambridge, VS: Academic Press), 133–148.

[ref13] Bateman A. O'Connell J. Lorenzini N. Gardner T. Fonagy P. (2016). A randomised controlled trial of mentalization-based treatment versus structured clinical management for patients with comorbid borderline personality disorder and antisocial personality disorder. BMC Psychiatry 16:304. doi: 10.1186/s12888-016-1000-9, PMID: 27577562 PMC5006360

[ref14] Beauchaine T.P. Hinshaw S.P. (2014). The Oxford handbook of externalizing spectrum disorders. Oxford, UK: Oxford University Press.

[ref15] Berg J. Dickhaut J. McCabe K. (1995). Trust, reciprocity, and social history. Games Econ. Behav. 10, 122–142. doi: 10.1006/game.1995.1027

[ref16] Bernstein D. P. Keulen-de Vos M. Clercx M. de Vogel V. Kersten G. C. M. Lancel M.. (2021). Schema therapy for violent PD offenders: a randomized clinical trial. Psychol. Med. 53, 88–102. doi: 10.1017/S0033291721001161, PMID: 34127158 PMC9874993

[ref17] Blair R. J. R. (2001). Neurocognitive models of aggression, the antisocial personality disorders, and psychopathy. J. Neurol. Neurosurg. Psychiat. 71, 727–731. doi: 10.1136/jnnp.71.6.727 11723191 PMC1737625

[ref18] Blair R. Colledge E. Murray L. Mitchell D. (2001). A selective impairment in the processing of sad and fearful expressions in children with psychopathic tendencies. J. Abnorm. Child Psychol. 29, 491–498. doi: 10.1023/A:1012225108281 11761283

[ref19] Blair K. S. Richell R. A. Mitchell D. G. V. Leonard A. Morton J. Blair R. J. R. (2006). They know the words, but not the music: affective and semantic priming in individuals with psychopathy. Biol. Psychol. 73, 114–123. doi: 10.1016/j.biopsycho.2005.12.006 16574302

[ref20] Blair R. J. R. Veroude K. Buitelaar J. K. (2018). Neuro-cognitive system dysfunction and symptom sets: a review of fMRI studies in youth with conduct problems. Neurosci. Biobehav. Rev. 91, 69–90. doi: 10.1016/j.neubiorev.2016.10.022 27794436

[ref21] Bowlby J. (1969). Attachment and Loss. New York: Basic Books.

[ref22] Bradshaw C. P. Garbarino J. (2006). Social cognition as a mediator of the influence of family and community violence on adolescent development: implications for intervention. Ann. N. Y. Acad. Sci. 1036, 85–105. doi: 10.1196/annals.1330.005, PMID: 15817732

[ref23] Brazil I. A. van Dongen J. D. M. Maes J. H. R. Mars R. B. Baskin-Sommers A. R. (2018). Classification and treatment of antisocial individuals: from behavior to biocognition. Neurosci. Biobehav. Rev. 91, 259–277. doi: 10.1016/j.neubiorev.2016.10.010 27760372

[ref24] Brennan G. M. Baskin-Sommers A. R. (2021). Cognitive mechanisms influencing facial emotion processing in psychopathy and externalizing. Personal. Disord. Theory Res. Treat. 12, 581–593. doi: 10.1037/per0000473 33301340

[ref25] Burt S. A. Donnellan M. (2009). Development and validation of the subtypes of antisocial behavior questionnaire. Aggress. Behav. 35, 376–398. doi: 10.1002/ab.20314, PMID: 19618380

[ref26] Burt S. A. Donnellan M. B. Tackett J. L. (2012). Should social aggression be considered “antisocial”? J. Psychopathol. Behav. Assess. 34, 153–163. doi: 10.1007/s10862-011-9267-0

[ref27] Burt S. A. Mikolajewski A. J. Larson C. L. (2009). Do aggression and rule-breaking have different interpersonal correlates? A study of antisocial behavior subtypes, negative affect, and hostile perceptions of others. Aggress. Behav. 35, 453–461. doi: 10.1002/ab.20324, PMID: 19780037

[ref28] Chakhssi F. Kersten T. de Ruiter C. Bernstein D. P. (2014). Treating the untreatable: a single case study of a psychopathic inpatient treated with Schema Therapy. Psychotherapy 51, 447–461. doi: 10.1037/a0035773 24684220

[ref29] Chang S.-A. A. Tillem S. Benson-Williams C. Baskin-Sommers A. (2021). Cognitive empathy in subtypes of antisocial individuals. Front. Psych. 12:677975. doi: 10.3389/fpsyt.2021.677975 PMC8287099 34290630

[ref30] Chapman H. Gillespie S. M. Mitchell I. J. (2018). Facial affect processing in incarcerated violent males: a systematic review. Aggress. Violent Behav. 38, 123–138. doi: 10.1016/j.avb.2017.10.006

[ref31] Chen P. Coccaro E. F. Lee R. Jacobson K. C. (2012). Moderating effects of childhood maltreatment on associations between social information processing and adult aggression. Psychol. Med. 42, 1293–1304. doi: 10.1017/S0033291711002212, PMID: 22008562 PMC4255557

[ref32] Cima M. Raine A. (2009). Distinct characteristics of psychopathy relate to different subtypes of aggression. Personal. Individ. Differ. 47, 835–840. doi: 10.1016/j.paid.2009.06.031

[ref33] Cleckley H. (1988). The mask of sanity. St. Louis, MO: Mosby. 10.1080/00325481.1951.11694097 14807904

[ref34] Costa P. McCrae R. (1992). Normal personality assessment in clinical practice: the NEO personality inventory. Psychol. Assess. 4, 5–13. doi: 10.1037/1040-3590.4.1.5

[ref35] Crowe S. L. Blair R. J. R. (2008). The development of antisocial behavior: what can we learn from functional neuroimaging studies? Dev. Psychopathol. 20, 1145–1159. doi: 10.1017/S0954579408000540 18838035

[ref36] Davidson K. M. Tyrer P. Tata P. Cooke D. Gumley A. Ford I.. (2009). Cognitive behaviour therapy for violent men with antisocial personality disorder in the community: an exploratory randomized controlled trial. Psychol. Med. 39, 569–577. doi: 10.1017/S0033291708004066 18667099

[ref37] Dawel A. O’Kearney R. McKone E. Palermo R. (2012). Not just fear and sadness: meta-analytic evidence of pervasive emotion recognition deficits for facial and vocal expressions in psychopathy. Neurosci. Biobehav. Rev. 36, 2288–2304. doi: 10.1016/j.neubiorev.2012.08.006 22944264

[ref38] De Castro B. O. Veerman J. W. Koops W. Bosch J. D. Monshouwer H. J. (2002). Hostile attribution of intent and aggressive behavior: a meta-analysis. Child Dev. 73, 916–934. doi: 10.1111/1467-8624.00447 12038560

[ref39] Decuyper M. De Pauw S. De Fruyt F. De Bolle M. De Clercq B. J. (2009). A meta-analysis of psychopathy-, antisocial PD- and FFM associations. Eur. J. Personal. 23, 531–565. doi: 10.1002/per.729

[ref40] Del Gaizo A. L. Falkenbach D. M. (2008). Primary and secondary psychopathic-traits and their relationship to perception and experience of emotion. Personal. Individ. Differ. 45, 206–212. doi: 10.1016/j.paid.2008.03.019

[ref41] Dodge K. A. Coie J. D. (1987). Social-information-processing factors in reactive and proactive aggression in children's peer groups. J. Pers. Soc. Psychol. 53, 1146–1158. doi: 10.1037/0022-3514.53.6.1146, PMID: 3694454

[ref42] Dodge K. A. Crick N. R. (1990). Social information-processing bases of aggressive behavior in children. Personal. Soc. Psychol. Bull. 16, 8–22. doi: 10.1177/0146167290161002

[ref43] Dolan M. Fullam R. (2004). Theory of mind and mentalizing ability in antisocial personality disorders with and without psychopathy. Psychol. Med. 34, 1093–1102. doi: 10.1017/S0033291704002028 15554579

[ref44] Dolan M. Fullam R. (2006). Face affect recognition deficits in personality-disordered offenders: association with psychopathy. Psychol. Med. 36, 1563–1569. doi: 10.1017/S0033291706008634, PMID: 16893483

[ref45] Dorrepaal E. Thomaes K. Draijer N. (2008). Vroeger en verder: stabilisatiecursus na een geschiedenis van misbruik of mishandeling. Amsterdam: Harcourt Assessment.

[ref46] Drayton L. A. Santos L. R. Baskin-Sommers A. (2018). Psychopaths fail to automatically take the perspective of others. Proc. Natl. Acad. Sci. U. S. A. 115, 3302–3307. doi: 10.1073/pnas.1721903115, PMID: 29531085 PMC5879707

[ref47] Engelmann J. B. Schmid B. De Dreu C. K. W. Chumbley J. Fehr E. (2019). On the psychology and economics of antisocial personality. Proc. Natl. Acad. Sci. 116, 12781–12786. doi: 10.1073/pnas.1820133116, PMID: 31186356 PMC6601288

[ref48] Erikson E.H. (1963). Childhood and society. New York: Norton.

[ref49] Fareri D. S. (2019). Neurobehavioral mechanisms supporting trust and reciprocity. Front. Hum. Neurosci. 13:271. doi: 10.3389/fnhum.2019.00271 31474843 PMC6705214

[ref50] Farkas L. Cyr M. Lebeau T. Lemay J. (2010). Effectiveness of MASTR/EMDR therapy for traumatized adolescents. J. Child Adolesc. Trauma 3, 125–142. doi: 10.1080/19361521003761325

[ref51] Fett A. K. Shergill S. S. Gromann P. M. Dumontheil I. Blakemore S. J. Yakub F.. (2014). Trust and social reciprocity in adolescence--a matter of perspective-taking. J. Adolesc. 37, 175–184. doi: 10.1016/j.adolescence.2013.11.011 24439623

[ref52] Flight J. I. Forth A. E. (2007). Instrumentally violent youths: the roles of psychopathic traits, empathy, and attachment. Crim. Justice Behav. 34, 739–751. doi: 10.1177/0093854807299462

[ref53] Fonagy P. Gergely G. Target M. (2007). The parent-infant dyad and the construction of the subjective self. J. Child Psychol. Psychiatry 48, 288–328. doi: 10.1111/j.1469-7610.2007.01727.x, PMID: 17355400

[ref54] Fonagy P. Levinson A. J. (2004). Offending and attachment: the relationship between interpersonal awareness and offending in a prison population with psychiatric disorder. Can. J. Psychoanal. 12, 225–251.

[ref55] Fonagy P. Luyten P. Allison E. (2015). Epistemic petrification and the restoration of epistemic trust: a new conceptualization of borderline personality disorder and its psychosocial treatment. J. Personal. Disord. 29, 575–609. doi: 10.1521/pedi.2015.29.5.575 26393477

[ref56] Fossati A. Acquarini E. Feeney J. Borroni S. Grazioli F. Giarolli L.. (2009). Alexithymia and attachment insecurities in impulsive aggression. Attach Hum. Dev. 11, 165–182. doi: 10.1080/14616730802625235 19266364

[ref57] Foster E. M. Jones D. E. (2005). The high costs of aggression: public expenditures resulting from conduct disorder. Am. J. Public Health 95, 1767–1772. doi: 10.2105/AJPH.2004.061424 16131639 PMC1449434

[ref58] Frick P. J. Dickens C. (2006). Current perspectives on conduct disorder. Curr. Psychiatry Rep. 8, 59–72. doi: 10.1007/s11920-006-0082-3 16513044

[ref59] Frick P. J. Ray J. V. (2015). Evaluating callous-unemotional traits as a personality construct. J. Pers. 83, 710–722. doi: 10.1111/jopy.12114 25039236

[ref60] Frick P. J. Ray J. V. Thornton L. C. Kahn R. E. (2014). Can callous-unemotional traits enhance the understanding, diagnosis, and treatment of serious conduct problems in children and adolescents? A comprehensive review. Psychol. Bull. 140, 1–57. doi: 10.1037/a0033076, PMID: 23796269

[ref61] Gibbon S. Khalifa N. R. Cheung N. H. Y. Völlm B. A. McCarthy L. (2020). Psychological interventions for antisocial personality disorder. Cochrane Database Syst. Rev. 2020:CD007668. doi: 10.1002/14651858.CD007668.pub3, PMID: 32880104 PMC8094166

[ref62] Gilbert P. Basran J. (2019). The evolution of prosocial and antisocial competitive behavior and the emergence of prosocial and antisocial leadership styles. Front. Psychol. 10:610. doi: 10.3389/fpsyg.2019.00610, PMID: 31293464 PMC6603082

[ref63] Gillespie S. M. Kongerslev M. T. Sharp C. Bo S. Abu-Akel A. M. (2018). Does affective theory of mind contribute to proactive aggression in boys with conduct problems and psychopathic tendencies? Child Psychiatry Hum. Dev. 49, 906–916. doi: 10.1007/s10578-018-0806-8, PMID: 29704083 PMC6208983

[ref64] Gillespie S. M. Lee J. Williams R. Jones A. (2022). Psychopathy and response inhibition: a meta-analysis of go/no-go and stop signal task performance. Neurosci. Biobehav. Rev. 142:104868. doi: 10.1016/j.neubiorev.2022.104868, PMID: 36113781

[ref65] Gray N. S. Weidacker K. Snowden R. J. (2019). Psychopathy and impulsivity: the relationship of psychopathy to different aspects of UPPS-P impulsivity. Psychiatry Res. 272, 474–482. doi: 10.1016/j.psychres.2018.12.155 30611967

[ref66] György G. Unoka Z. (2008). “Attachment, affect-regulation, and mentalization: The developmental origins of the representational affective self’’ in *Social cognition and developmental psychopathology*. eds. C. Sharp, P. Fonagy and I. Goodyer (Oxford, UK: Oxford University Press), 305–342.

[ref67] Hammond C. J. Potenza M. N. Mayes L. C. (2011). “Development of impulse control, inhibition, and self-regulatory behaviors in normative populations across the lifespan,” in The Oxford Handbook of Impulse Control Disorders. eds. Grant J. E. Potenza M. N. (Oxford: Oxford University Press)

[ref68] Hicklin J. Widiger T. A. (2005). Similarities and differences among antisocial and psychopathic self-report inventories from the perspective of general personality functioning. Eur. J. Personal. 19, 325–342. doi: 10.1002/per.562

[ref69] Hicks B. M. Markon K. E. Patrick C. J. Krueger R. F. Newman J. P. (2004). Identifying psychopathy subtypes on the basis of personality structure. Psychol. Assess. 16, 276–288. doi: 10.1037/1040-3590.16.3.276 15456383

[ref70] Hopwood C. J. Bagby R. M. Gralnick T. Ro E. Ruggero C. Mullins-Sweatt S.. (2020). Integrating psychotherapy with the hierarchical taxonomy of psychopathology (HiTOP). J. Psychother. Integr. 30, 477–497. doi: 10.1037/int0000156

[ref71] Hyde L. W. Waller R. Trentacosta C. J. Shaw D. S. Neiderhiser J. M. Ganiban J. M.. (2016). Heritable and nonheritable pathways to early callous-unemotional behaviors. Am. J. Psychiatry 173, 903–910. doi: 10.1176/appi.ajp.2016.15111381, PMID: 27056607 PMC5008992

[ref72] Ibáñez M. I. Sabater-Grande G. Barreda-Tarrazona I. Mezquita L. López-Ovejero S. Villa H.. (2016). Take the money and run: psychopathic behavior in the trust game. Front. Psychol. 7:1866. doi: 10.3389/fpsyg.2016.01866 27965606 PMC5125304

[ref73] Insel T. Cuthbert B. Garvey M. Heinssen R. Pine D. S. Quinn K.. (2010). Research domain criteria (RDoC): toward a new classification framework for research on mental disorders. Am. J. Psychiatry 167, 748–751. doi: 10.1176/appi.ajp.2010.09091379 20595427

[ref74] Jones Bartoli A. Forster A. Skuse D. (2007). What do you think you're looking at? Investigating social cognition in young offenders. Crim. Behav. Ment. Health 17, 101–106. doi: 10.1002/cbm.641 17295200

[ref75] Jurjako M. Malatesti L. Brazil I. A. (2020). Biocognitive classification of antisocial individuals without explanatory reductionism. Perspect. Psychol. Sci. 15, 957–972. doi: 10.1177/1745691620904160 32502369

[ref76] Kiehl K. A. Buckholtz J. W. (2010). Inside the mind of a psychopath. Sci. Am. Mind 21, 22–29. doi: 10.1038/scientificamericanmind0910-22

[ref77] Klein Tuente S. Bogaerts S. Bulten E. Keulen-de Vos M. Vos M. Bokern H.. (2020). Virtual reality aggression prevention therapy (VRAPT) versus waiting list control for forensic psychiatric inpatients: a multicenter randomized controlled trial. J. Clin. Med. 9:2258. doi: 10.3390/jcm9072258 32708637 PMC7409015

[ref78] Klein Tuente S. Bogaerts S. Veling W. (2019). Hostile attribution bias and aggression in adults - a systematic review. Aggress. Violent Behav. 46, 66–81. doi: 10.1016/j.avb.2019.01.009

[ref79] Kosson D. S. Lorenz A. R. Newman J. P. (2006). Effects of comorbid psychopathy on criminal offending and emotion processing in male offenders with antisocial personality disorder. J. Abnorm. Psychol. 115, 798–806. doi: 10.1037/0021-843X.115.4.798 17100537

[ref80] Kotov R. Krueger R. F. Watson D. Achenbach T. M. Althoff R. R. Bagby R. M.. (2017). The hierarchical taxonomy of psychopathology (HiTOP): a dimensional alternative to traditional nosologies. J. Abnorm. Psychol. 126, 454–477. doi: 10.1037/abn0000258 28333488

[ref81] Kraanen F. L. (2020). “Antisociale persoonlijkheidsstoornis en middelengebruik,” in Praktijkboek antisociaal gedrag en persoonlijkheidsproblematiek. eds. Rijckmans M. J. N. Van Dam A. Van den Bosch L. M. C. (Houten, NL: Bohn Stafleu van Loghum), 157–180.

[ref82] Krueger R. F. Hobbs K. A. Conway C. C. Dick D. M. Dretsch M. N. Eaton N. R.. (2021). Validity and utility of hierarchical taxonomy of psychopathology (HiTOP): II. Externalizing superspectrum. World Psychiatry 20, 171–193. doi: 10.1002/wps.20844, PMID: 34002506 PMC8129870

[ref83] Krueger R. F. Markon K. E. (2014). The role of the DSM-5 personality trait model in moving toward a quantitative and empirically based approach to classifying personality and psychopathology. Annu. Rev. Clin. Psychol. 10, 477–501. doi: 10.1146/annurev-clinpsy-032813-153732 24329179

[ref84] Leone J. M. Johnson M. P. Cohan C. L. Lloyd S. E. (2004). Consequences of male partner violence for low-income minority women. J. Marriage Fam. 66, 472–490. doi: 10.1111/j.1741-3737.2004.00032.x

[ref85] Lobbestael J. Arntz A. Cima M. Chakhssi F. (2009). Effects of induced anger in patients with antisocial personality disorder. Psychol. Med. 39, 557–568. doi: 10.1017/S0033291708005102 19171078

[ref86] Lobbestael J. Arntz A. Sieswerda S. (2005). Schema modes and childhood abuse in borderline and antisocial personality disorders. J. Behav. Ther. Exp. Psychiatry 36, 240–253. doi: 10.1016/j.jbtep.2005.05.006, PMID: 15953584

[ref87] Lobbestael J. Cima M. Arntz A. (2013). The relationship between adult reactive and proactive aggression, hostile interpretation bias, and antisocial personality disorder. J. Personal. Disord. 27, 53–66. doi: 10.1521/pedi.2013.27.1.53 23342957

[ref88] Loney B. R. Frick P. J. Clements C. B. Ellis M. L. Kerlin K. (2003). Callous-unemotional traits, impulsivity, and emotional processing in adolescents with antisocial behavior problems. J. Clin. Child Adolesc. Psychol. 32, 66–80. doi: 10.1207/S15374424JCCP3201_07 12573933

[ref89] Luyten P. Fonagy P. (2015). The neurobiology of mentalizing. Personal. Disord. Theory Res. Treat. 6:366. doi: 10.1037/per0000117 26436580

[ref90] Lynam D. Miller J. (2019). The basic trait of antagonism: an unfortunately underappreciated construct. J. Res. Pers. 81, 118–126. doi: 10.1016/j.jrp.2019.05.012

[ref91] Maneiro L. Gómez-Fraguela J. A. Cutrín O. Romero E. (2017). Impulsivity traits as correlates of antisocial behaviour in adolescents. Personal. Individ. Differ. 104, 417–422. doi: 10.1016/j.paid.2016.08.045

[ref92] Mann F. D. Briley D. A. Tucker-Drob E. M. Paige Harden K. (2015). A behavioral genetic analysis of callous-unemotional traits and big five personality in adolescence. J. Abnorm. Psychol. 124, 982–993. doi: 10.1037/abn0000099, PMID: 26595476 PMC5225906

[ref93] Marsden J. Glazebrook C. Tully R. Völlm B. (2019). Do adult males with antisocial personality disorder (with and without co-morbid psychopathy) have deficits in emotion processing and empathy? A systematic review. Aggress. Violent Behav. 48, 197–217. doi: 10.1016/j.avb.2019.08.009

[ref94] Marsh A. Blair R. (2008). Marsh AA, RJR Blair. Deficits in facial affect recognition among antisocial populations: a meta-analysis. Neurosci. Biobehav. Rev. 32, 454–465. doi: 10.1016/j.neubiorev.2007.08.003, PMID: 17915324 PMC2255599

[ref95] McMullen J. O'Callaghan P. Shannon C. Black A. Eakin J. (2013). Group trauma-focused cognitive-behavioural therapy with former child soldiers and other war-affected boys in the DR Congo: a randomised controlled trial. J. Child Psychol. Psychiatry 54, 1231–1241. doi: 10.1111/jcpp.12094, PMID: 23738530

[ref96] Milich R. Dodge K. A. (1984). Social information processing in child psychiatric populations. J. Abnorm. Child Psychol. 12, 471–489. doi: 10.1007/BF00910660 6747124

[ref97] Miller J. D. Lynam D. (2001). Structural models of personality and their relation to antisocial behavior: a META-analytic review*. Criminology 39, 765–798. doi: 10.1111/j.1745-9125.2001.tb00940.x

[ref98] Miller J. Lynam D. (2006). Reactive and proactive aggression: similarities and differences. Personal. Individ. Differ. 41, 1469–1480. doi: 10.1016/j.paid.2006.06.004

[ref99] Miller J. D. Lynam D. R. Jones S. (2008). Externalizing behavior through the lens of the five-factor model: a focus on agreeableness and conscientiousness. J. Pers. Assess. 90, 158–164. doi: 10.1080/00223890701845245, PMID: 18444110

[ref100] Miller J. D. Lynam D. R. Widiger T. A. Leukefeld C. (2001). Personality disorders as extreme variants of common personality dimensions: can the Five-Factor Model adequately represent psychopathy? J. Pers. 69, 253–276. 11339798 10.1111/1467-6494.00144

[ref101] Miller J. D. Zeichner A. Wilson L. F. (2012). Personality correlates of aggression: evidence from measures of the five-factor model, UPPS model of impulsivity, and BIS/BAS. J. Interpers. Violence 27, 2903–2919. doi: 10.1177/0886260512438279 22610830

[ref102] Moffitt T. E. (2018). Male antisocial behaviour in adolescence and beyond. Nat. Hum. Behav. 2, 177–186. doi: 10.1038/s41562-018-0309-4 30271880 PMC6157602

[ref103] Mokros A. Hare R. D. Neumann C. S. Santtila P. Habermeyer E. Nitschke J. (2015). Variants of psychopathy in adult male offenders: a latent profile analysis. J. Abnorm. Psychol. 124, 372–386. doi: 10.1037/abn0000042 25643206

[ref104] Moller C. Larsson M. H. Holmqvist R. Falkenstrom F. (2014). Mentalizing in young offenders. Psychoanal. Psychol. 31, 84–99. doi: 10.1037/a0035555

[ref105] Montagne B. van Honk J. Kessels R. P. C. Frigerio E. Burt M. van Zandvoort M. J. E.. (2005). Reduced efficiency in recognising fear in subjects scoring high on psychopathic personality characteristics. Personal. Individ. Differ. 38, 5–11. doi: 10.1016/j.paid.2004.02.008

[ref106] Moore A. A. Blair R. J. Hettema J. M. Roberson-Nay R. (2019). The genetic underpinnings of callous-unemotional traits: a systematic research review. Neurosci. Biobehav. Rev. 100, 85–97. doi: 10.1016/j.neubiorev.2019.02.018 30817934 PMC6756755

[ref107] Münch R. Walter H. Müller S. (2020). Should behavior harmful to others be a sufficient criterion of mental disorders? Conceptual problems of the diagnoses of antisocial personality disorder and pedophilic disorder. Front. Psych. 11:558655. doi: 10.3389/fpsyt.2020.558655, PMID: 33093836 PMC7523554

[ref108] Muratori P. Lochman J. E. Lai E. Milone A. Nocentini A. Pisano S.. (2016). Which dimension of parenting predicts the change of callous unemotional traits in children with disruptive behavior disorder? Compr. Psychiatry 69, 202–210. doi: 10.1016/j.comppsych.2016.06.002 27423362

[ref109] Newbury-Helps J. Feigenbaum J. Fonagy P. (2017). Offenders with antisocial personality disorder display more impairments in Mentalizing. J. Personal. Disord. 31, 232–255. doi: 10.1521/pedi_2016_30_246, PMID: 27064853

[ref110] Nowak M. A. (2006). Five rules for the evolution of cooperation. Science 314, 1560–1563. doi: 10.1126/science.1133755, PMID: 17158317 PMC3279745

[ref111] Ogle C. M. Siegler I. C. Beckham J. C. Rubin D. C. (2017). Neuroticism increases PTSD symptom severity by amplifying the emotionality, rehearsal, and centrality of trauma memories. J. Pers. 85, 702–715. doi: 10.1111/jopy.12278 27517170 PMC6196079

[ref112] Ostrom E. Walker J. (2003). Trust and Reciprocity: Interdisciplinary Lessons for Experimental Research. New York, NY Russell Sage Foundation.

[ref113] Poulin F. Boivin M. (2000). Reactive and proactive aggression: evidence of a two-factor model. Psychol. Assess. 12, 115–122. doi: 10.1037/1040-3590.12.2.115, PMID: 10887757

[ref114] Poythress N. G. Edens J. F. Skeem J. L. Lilienfeld S. O. Douglas K. S. Frick P. J.. (2010). Identifying subtypes among offenders with antisocial personality disorder: a cluster-analytic study. J. Abnorm. Psychol. 119, 389–400. doi: 10.1037/a0018611 20455611

[ref115] Protic S. Wittmann L. Taubner S. Dimitrijevic A. (2020). Differences in attachment dimensions and reflective functioning between traumatized juvenile offenders and maltreated non-delinquent adolescents from care services. Child Abuse Negl. 103:104420. doi: 10.1016/j.chiabu.2020.104420, PMID: 32146268

[ref116] Puiu A. A. Wudarczyk O. Goerlich K. S. Votinov M. Herpertz-Dahlmann B. Turetsky B.. (2018). Impulsive aggression and response inhibition in attention-deficit/hyperactivity disorder and disruptive behavioral disorders: findings from a systematic review. Neurosci. Biobehav. Rev. 90, 231–246. doi: 10.1016/j.neubiorev.2018.04.016, PMID: 29689282

[ref117] Raine A. Dodge K. Loeber R. Gatzke-Kopp L. Lynam D. Reynolds C.. (2006). The reactive-proactive aggression questionnaire: differential correlates of reactive and proactive aggression in adolescent boys. Aggress. Behav. 32, 159–171. doi: 10.1002/ab.20115, PMID: 20798781 PMC2927832

[ref118] Reardon K. W. Tackett J. L. Lynam D. (2018). The personality context of relational aggression: a Five-Factor Model profile analysis. Personal. Disord. Theory Res. Treat. 9, 228–238. doi: 10.1037/per0000231 28095002

[ref119] Ricci R. J. Clayton C. A. Shapiro F. (2006). Some effects of EMDR on previously abused child molesters: theoretical reviews and preliminary findings. J. Forensic Psychiatry Psychol. 17, 538–562. doi: 10.1080/14789940601070431

[ref120] Richey A. Brown S. Fite P. J. Bortolato M. (2016). The role of hostile attributions in the associations between child maltreatment and reactive and proactive aggression. J. Aggress. Maltreat. Trauma 25, 1043–1057. doi: 10.1080/10926771.2016.1231148, PMID: 29386881 PMC5788315

[ref121] Ripley A. J. Clapp J. D. Wilkowski B. M. (2019). PTSD and anger: evaluation of an indirect effect model in a civilian trauma sample. J. Behav. Ther. Exp. Psychiatry 64, 149–157. doi: 10.1016/j.jbtep.2019.02.004 31035245

[ref122] Rodriguez-Seijas C. Ruggero C. Eaton N. R. Krueger R. F. (2019). The DSM-5 alternative model for personality disorders and clinical treatment: a review. Curr. Treat. Opt. Psychiatry 6, 284–298. doi: 10.1007/s40501-019-00187-7

[ref123] Rokop J. DiCiro M. Sreenivasan S. Weinberger L. E. (2021). A dimensional framework for the use of ASPD in SVP civil commitment. J. Forensic Psychiatry Psychol. 32, 155–179. doi: 10.1080/14789949.2020.1836251

[ref124] Roose A. Bijttebier P. Claes L. Lilienfeld S. O. De Fruyt F. Decuyper M. (2012). Psychopathic traits in adolescence and the five factor model of personality. J. Psychopathol. Behav. Assess. 34, 84–93. doi: 10.1007/s10862-011-9243-8

[ref125] Rosenberg H. J. Rosenberg S. D. Vance J. E. Wolford G. L. Ashley S. W. Howard M. L. (2014). Trauma exposure, psychiatric disorders, and resiliency in juvenile-justice-involved youth. Psychol. Trauma Theory Res. Pract. Policy 6, 430–437. doi: 10.1037/a0033199

[ref126] Ruiz M. A. Pincus A. L. Schinka J. A. (2008). Externalizing pathology and the five-factor model: a meta-analysis of personality traits associated with antisocial personality disorder, substance use disorder, and their co-occurrence. J. Personal. Disord. 22, 365–388. doi: 10.1521/pedi.2008.22.4.365, PMID: 18684050

[ref127] Sargeant M. N. Daughters S. B. Curtin J. J. Schuster R. Lejuez C. W. (2011). Unique roles of antisocial personality disorder and psychopathic traits in distress tolerance. J. Abnorm. Psychol. 120, 987–992. doi: 10.1037/a0024161 21668082 PMC3229260

[ref128] Saylor K. E. Amann B. H. (2016). Impulsive aggression as a comorbidity of attention-deficit/hyperactivity disorder in children and adolescents. J. Child Adolesc. Psychopharmacol. 26, 19–25. doi: 10.1089/cap.2015.0126 26744906 PMC4779282

[ref129] Schonenberg M. Jusyte A. (2014). Investigation of the hostile attribution bias toward ambiguous facial cues in antisocial violent offenders. Eur. Arch. Psychiatry Clin. Neurosci. 264, 61–69. doi: 10.1007/s00406-013-0440-1, PMID: 23990116

[ref130] Schwartz D. Dodge K. A. Coie J. D. Hubbard J. A. Cillessen A. H. N. Lemerise E. A.. (1998). Social-cognitive and behavioral correlates of aggression and victimization in boys' play groups. J. Abnorm. Child Psychol. 26, 431–440. doi: 10.1023/A:1022695601088, PMID: 9915650

[ref131] Sebastian C. L. McCrory E. J. P. Cecil C. A. M. Lockwood P. L. De Brito S. A. Fontaine N. M. G.. (2012). Neural responses to affective and cognitive theory of mind in children with conduct problems and varying levels of callous-unemotional traits. Arch. Gen. Psychiatry 69, 814–822. doi: 10.1001/archgenpsychiatry.2011.2070, PMID: 22868935

[ref132] Serie C. Tilburg C. van Dam A. De Ruiter C. (2015). Agressieregulatiegroepstherapie voor relationeel geweldplegers – Een open trial. Gedragstherapie 48, 265–281.

[ref133] Shamay Tsoory S. G. Harari H. Aharon-Peretz J. Levkovitz Y. (2010). The role of the orbitofrontal cortex in affective theory of mind deficits in criminal offenders with psychopathic tendencies. Cortex 46, 668–677. doi: 10.1016/j.cortex.2009.04.008, PMID: 19501818

[ref134] Simpson J. (2007). “Foundations of interpersonal trust,” in Social Psychology: Handbook of Basic Principles. eds. Kruglanski A. W. Higgins E. T. (New York, NY: The Guilford Press), 587–607.

[ref135] Skodol A. E. Morey L. C. Bender D. S. Oldham J. M. (2015). The alternative DSM-5 model for personality disorders: a clinical application. Am. J. Psychiatry 172, 606–613. doi: 10.1176/appi.ajp.2015.14101220 26130200

[ref136] Smeijers D. Rinck M. Bulten E. van den Heuvel T. Verkes R. J. (2017). Generalized hostile interpretation bias regarding facial expressions: characteristic of pathological aggressive behavior. Aggress. Behav. 43, 386–397. doi: 10.1002/ab.21697 28191653

[ref137] Smith M. E. (2011). A qualitative review of perception of change for male perpetrators of domestic abuse following abuser schema therapy (AST). Couns. Psychother. Res. 11, 156–164. doi: 10.1080/14733145.2010.486863

[ref138] Soberman G. B. Greenwald R. Rule D. L. (2002). A controlled study of eye movement desensitization and reprocessing (EMDR) for boys with conduct problem. J. Aggress. Maltreat. Trauma 6, 217–236. doi: 10.1300/J146v06n01_11

[ref139] Stimmel M. A. Cruise K. R. Ford J. D. Weiss R. A. (2014). Trauma exposure, posttraumatic stress disorder symptomatology, and aggression in male juvenile offenders. Psychol. Trauma Theory Res. Pract. Policy 6, 184–191. doi: 10.1037/a0032509

[ref140] Sun L. Niu G. Li J. Du H. Hu X. Yang S.. (2020). Trait aggression affects the response inhibition to angry expressions: an event-related brain potential study. Personal. Individ. Differ. 152:109553. doi: 10.1016/j.paid.2019.109553

[ref141] Sun J.-W. Xue J.-M. Bai H.-Y. Zhang H.-H. Lin P.-Z. Cao F.-L. (2016). The association between negative life events, neuroticism and aggression in early adulthood. Personal. Individ. Differ. 102, 139–144. doi: 10.1016/j.paid.2016.06.066

[ref142] Thielmann I. Hilbig B. E. (2015). Trust: an integrative review from a person–situation perspective. Rev. Gen. Psychol. 19, 249–277. doi: 10.1037/gpr0000046

[ref143] Thylstrup B. Hesse M. Schrøder S. (2015). Psycho-education for substance use and antisocial personality disorder: a randomized trial. BMC Psychiatry 15:283. doi: 10.1186/s12888-015-0661-0 26573140 PMC4647713

[ref144] Thylstrup B. Schrøder S. Fridell M. Hesse M. (2017). Did you get any help? A post-hoc secondary analysis of a randomized controlled trial of psychoeducation for patients with antisocial personality disorder in outpatient substance abuse treatment programs. BMC Psychiatry 17, 1–10. doi: 10.1186/s12888-016-1165-2 28068951 PMC5223491

[ref145] Trivers R. L. (1971). The evolution of reciprocal altruism. Q. Rev. Biol. 46, 35–57.

[ref146] Turner H. A. Finkelhor D. Ormrod R. (2010). The effects of adolescent victimization on self-concept and depressive symptoms. Child Maltreat. 15, 76–90. doi: 10.1177/1077559509349444 19926630

[ref147] Tuvblad C. Beaver K. M. (2013). Genetic and environmental influences on antisocial behavior. J. Crim. Just. 41, 273–276. doi: 10.1016/j.jcrimjus.2013.07.007, PMID: 24526799 PMC3920596

[ref148] Tuvblad C. Raine A. Zheng M. Baker L. A. (2009). Genetic and environmental stability differs in reactive and proactive aggression. Aggress. Behav. 35, 437–452. doi: 10.1002/ab.20319, PMID: 19688841 PMC2771207

[ref149] Van Dam A. Rijckmans M. J. N. (2020). “Antisociaal gedrag bij psychische stoornissen: diagnostiek, betekenis en risico,” in Praktijkboek antisociaal gedrag en persoonlijkheidsproblematiek [A practitioners guide for antisocial behavior and personality disorders]. eds. Rijckmans M. J. N. Van Dam A. Van den Bosch L. M. C. (Houten: Bohn Stafleuk van Loghum), 13–34.

[ref150] Van Dam A. Rijckmans M. Bosch L. (2022). Explaining the willingness of clinicians to work with patients with antisocial personality disorder using the theory of planned behaviour and emotional reactions. Clin. Psychol. Psychother. 29, 676–686. doi: 10.1002/cpp.2661 34433227 PMC9292584

[ref151] Van Dam A. van Tilburg C.A. Steenkist P. Buisman M. (2012). Niet meer door het lint [not loosing it anymore]. Utrecht: Bohn Stafleu van Loghum.

[ref152] Van den Bosch L. M. C. Rijckmans M. J. N. Decoene S. Chapman A. L. (2018). Treatment of antisocial personality disorder: development of a practice focused framework. Int. J. Law Psychiatry 58, 72–78. doi: 10.1016/j.ijlp.2018.03.002, PMID: 29853015

[ref153] Van Doesum N. van Lange D. Lange P. (2013). Social mindfulness: skill and will to navigate the social world. J. Pers. Soc. Psychol. 105, 86–103. doi: 10.1037/a0032540, PMID: 23647176

[ref154] Van Tilburg C. A. (2020). “Behandeling van trauma bij de antisociale persoonlijkheidsstoornis,” in Praktijkboek antisociaal gedrag en persoonlijkheidsproblematiek [practitioners guide for antisocial behavior and personality disorders]. eds. Rijckmans M. J. N. van Dam A. van den Bosch L. M. C. (Houten: Bohn Stafleu van Loghum), 205–235.

[ref155] Velotti P. Garofalo C. Dimaggio G. Fonagy P. (2019). Mindfulness, alexithymia, and empathy moderate relations between trait aggression and antisocial personality disorder traits. Mindfulness 10, 1082–1090. doi: 10.1007/s12671-018-1048-3

[ref156] Venables N. C. Hall J. R. Patrick C. J. (2014). Differentiating psychopathy from antisocial personality disorder: a triarchic model perspective. Psychol. Med. 44, 1005–1013. doi: 10.1017/S003329171300161X, PMID: 23834781

[ref157] Vize C. E. Collison K. L. Miller J. D. Lynam D. R. (2019). Using Bayesian methods to update and expand the meta-analytic evidence of the five-factor model's relation to antisocial behavior. Clin. Psychol. Rev. 67, 61–77. doi: 10.1016/j.cpr.2018.09.001, PMID: 30292437

[ref158] Volkert J. Gablonski T. C. Rabung S. (2018). Prevalence of personality disorders in the general adult population in Western countries: systematic review and meta-analysis. Br. J. Psychiatry J. Ment. Sci. 213, 709–715. doi: 10.1192/bjp.2018.202 30261937

[ref159] Waller R. Hyde L. W. Klump K. L. Burt S. A. (2018). Parenting is an environmental predictor of callous-unemotional traits and aggression: a monozygotic twin differences study. J. Am. Acad. Child Adolesc. Psychiatry 57, 955–963. doi: 10.1016/j.jaac.2018.07.882, PMID: 30522741 PMC6296820

[ref160] Ware A. Wilson C. Tapp J. Moore E. (2016). Mentalisation-based therapy (MBT) in a high-secure hospital setting: expert by experience feedback on participation. J. Forensic Psychiatry Psychol. 27, 722–744. doi: 10.1080/14789949.2016.1174725

[ref161] Waugh M. H. Hopwood C. J. Krueger R. F. Morey L. C. Pincus A. L. Wright A. G. C. (2017). Psychological assessment with the DSM-5 alternative model for personality disorders: tradition and innovation. Prof. Psychol. Res. Pr. 48, 79–89. doi: 10.1037/pro0000071, PMID: 28450760 PMC5403154

[ref162] Wetterborg D. Dehlbom P. Langstrom N. Andersson G. Fruzzetti A. E. Enebrink P. (2020). Dialectical behavior therapy for men with borderline personality disorder and antisocial behavior: a clinical trial. J. Personal. Disord. 34, 22–39. doi: 10.1521/pedi_2018_32_379, PMID: 30355023

[ref163] White S. F. VanTieghem M. Brislin S. J. Sypher I. Sinclair S. Pine D. S.. (2016). Neural correlates of the propensity for retaliatory behavior in youth with disruptive behavior disorders. Am. J. Psychiatry 173, 282–290. doi: 10.1176/appi.ajp.2015.15020250 26441155 PMC4950844

[ref164] Widiger T. A. Samuel D. B. (2005). Diagnostic categories or dimensions? A question for the diagnostic and statistical manual of mental disorders–fifth edition. J. Abnorm. Psychol. 114, 494–504. doi: 10.1037/0021-843x.114.4.494 16351373

[ref165] Wilson K. Juodis M. Porter S. (2011). Fear and loathing in psychopaths: a meta-analytic investigation of the facial affect recognition deficit. Crim. Justice Behav. 38, 659–668. doi: 10.1177/0093854811404120

[ref166] Wong S. C. P. Gordon A. (2013). The violence reduction programme: a treatment programme for violence-prone forensic clients. Psychol. Crime Law 19, 461–475. doi: 10.1080/1068316X.2013.758981

[ref167] Wong S. C. P. Gordon A. Gu D. Lewis K. Olver M. E. (2012). The effectiveness of violence reduction treatment for psychopathic offenders: empirical evidence and a treatment model. Int. J. Forensic Ment. Health 11, 336–349. doi: 10.1080/14999013.2012.746760

[ref168] Woodworth M. Porter S. (2002). In cold blood: characteristics of criminal homicides as a function of psychopathy. J. Abnorm. Psychol. 111, 436–445. doi: 10.1037/0021-843X.111.3.436, PMID: 12150419

[ref169] Woodworth M. Waschbusch D. (2008). Emotional processing in children with conduct problems and callous/unemotional traits. Child Care Health Dev. 34, 234–244. doi: 10.1111/j.1365-2214.2007.00792.x 18028474

[ref170] Wright S. A. Russell M. C. (2013). Treating violent impulses: a case study utilizing eye movement desensitization and reprocessing with a military client. Clin. Case Stud. 12, 128–144. doi: 10.1177/1534650112469461

[ref171] Wu J. Balliet D. P. Tybur J. M. Arai S. van Lange P. A. M. Yamagishi T. (2017). Life history strategy and human cooperation in economic games. Evol. Hum. Behav. 38, 496–505. doi: 10.1016/j.evolhumbehav.2017.03.002

[ref172] Young J.E. Klosko J.S. Weishaar M.E. (2003). Schema Therapy: A Practitioner's Guide. New York: Guilford Publications.

[ref173] Zhu W. Chen Y. Xia L.-X. (2020). Childhood maltreatment and aggression: the mediating roles of hostile attribution bias and anger rumination. Personal. Individ. Differ. 162:110007. doi: 10.1016/j.paid.2020.110007

